# Chia seeds as a potential cognitive booster in the APP23 Alzheimer’s disease model

**DOI:** 10.1038/s41598-020-75209-z

**Published:** 2020-10-26

**Authors:** Stefanie Schreyer, Charlotte Klein, Anna Pfeffer, Justyna Rasińska, Laura Stahn, Karlotta Knuth, Basim Abuelnor, Alina Elisabeth Catharina Panzel, André Rex, Stefan Koch, Shabnam Hemmati-Sadeghi, Barbara Steiner

**Affiliations:** 1 Department of Neurology, Charité-Universitätsmedizin Berlin, Freie Universität Berlin, Humboldt-Universität Zu Berlin, and Berlin Institute of Health, Charitéplatz 1, 10117 Berlin, Germany; 2 Department of Experimental Neurology and Center for Stroke Research Berlin, Charité-Universitätsmedizin Berlin, Freie Universität Berlin, Humboldt-Universität Zu Berlin, and Berlin Institute of Health, Charitéplatz 1, 10117 Berlin, Germany; 3 NeuroCure Cluster of Excellence and Charité Core Facility 7T Experimental MRIs, Charité-Universitätsmedizin Berlin, Freie Universität Berlin, Humboldt-Universität Zu Berlin, and Berlin Institute of Health, Charitéplatz 1, 10117 Berlin, Germany

**Keywords:** Neuroscience, Diseases, Endocrinology, Neurology, Pathogenesis, Risk factors

## Abstract

Glucose hypometabolism potentially contributes to Alzheimer’s disease (AD) and might even represent an underlying mechanism. Here, we investigate the relationship of diet-induced metabolic stress and AD as well as the therapeutic potential of chia seeds as a modulator of glucose metabolism in the APP23 mouse model. 4–6 (pre-plaque stage, PRE) and 28–32 (advanced-plaque stage, ADV) weeks old APP23 and wild type mice received pretreatment for 12 weeks with either sucrose-rich (SRD) or control diet, followed by 8 weeks of chia seed supplementation. Although ADV APP23 mice generally showed functioning glucose homeostasis, they were more prone to SRD-induced glucose intolerance. This was accompanied by elevated corticosterone levels and mild insulin insensitivity. Chia seeds improved spatial learning deficits but not impaired cognitive flexibility, potentially mediated by amelioration of glucose tolerance, attenuation of corticosterone levels and reversal of SRD-induced elevation of pro-inflammatory cytokine levels. Since cognitive symptoms and plaque load were not aggravated by SRD-induced metabolic stress, despite enhanced neuroinflammation in the PRE group, we conclude that impairments of glucose metabolism do not represent an underlying mechanism of AD in this mouse model. Nevertheless, chia seeds might provide therapeutic potential in AD as shown by the amelioration of cognitive symptoms.

## Introduction

Alzheimer’s disease (AD), affecting about 50 million people worldwide, is the most common form of dementia^1^. Pathological hallmarks of AD are extracellular amyloid beta (Aβ) plaques, neurofibrillary tangles of hyperphosphorylated tau protein and chronic neuroinflammation mediated by activated microglia, potentially further promoting disease progression^2^. Cognitive decline, induced by neurodegeneration, is the most apparent symptom of AD^2^. The hippocampus is very early affected during AD pathogenesis^3^. Alterations of adult hippocampal neurogenesis (AHN) have been shown in animal models and AD patients and might contribute to cognitive symptoms due to a loss of neuroplasticity^4^. Owing to the unknown underlying mechanisms of AD, currently no curative therapies are available.

So far, AD research focused on the amyloid cascade hypothesis, stating that the accumulation of Aβ, cleaved from the amyloid precursor protein (APP), and its formation into Aβ plaques is the underlying mechanism of cognitive decline^5^. However, the majority of clinical trials reducing Aβ burden with antibodies have shown no clinically significant improvements of cognitive performance^6^. Therefore, alternative potential causes, such as cerebral glucose metabolism, have been focused on recently. Cerebral glucose metabolism is reduced in AD patients, resulting in glucose intolerance and insulin resistance^7^. Glucose hypometabolism already occurs years before the onset of clinical symptoms of AD^7^, thus representing a potential underlying mechanism. Nevertheless, the implication of glucose hypometabolism in AD raises the question of causality. On the one hand, Aβ alters glucose metabolism, e.g. by reducing glucose uptake into cells^8^. On the other hand, glucose hypometabolism leads to energy deficiency and oxidative stress, which in turn induce Aβ overproduction^9^. Glucose hypometabolism in AD brains is similar to pathogenic changes occurring during type 2 diabetes mellitus (T2DM)^10^. Additionally, T2DM is a major risk factor for developing AD and T2DM patients often show symptoms of cognitive decline, which might be partially mediated by peripheral and central inflammation^11^. Another aspect linking AD to glucose metabolism is the dysregulation of the hypothalamic–pituitary–adrenal (HPA) axis, leading to alterations in glucocorticoid (GC) levels^12^. GCs act as functional antagonists of insulin, whereupon chronically elevated levels of GCs cause insulin resistance and glucose intolerance^13^. Elevated GC levels observed in AD patients correlate with hippocampal degeneration and cognitive decline^14^. Similarly, GC secretion is elevated in T2DM patients^15^.

Chia seeds (*Salvia hispanica* L.) are a low glycemic index food due to their low amount of carbohydrates compared to traditional grains, which is useful for glycemic control in diabetes^16,17^. The fat content of chia seeds contains a great proportion of essential omega-3 and omega-6 polyunsaturated fatty acids (PUFAs), such as the omega-3 PUFA α-linolenic acid (ALA)^18,19^. ALA is the precursor of eicosapentaenoic acid (EPA) and docosahexaenoic acid (DHA)^20^. Both, EPA and DHA are crucial for numerous brain functions such as signal transduction and exert neuroprotective effects like the reduction of neuroinflammation^21^. Furthermore, alterations in PUFA metabolism occur in AD patients^22^. Studies in diabetic rats have shown that chia seeds normalize insulin resistance and reduce visceral adiposity as well as cardiac and hepatic inflammation^23,24^. Likewise, human studies have shown the potential of chia seeds to reduce blood pressure, to promote weight loss, to stabilize blood glucose in diabetes and to increase serum omega-3 PUFAs^25^. However, to our knowledge no study has examined the effect of chia seeds on cognitive functions in healthy or diseased humans. Only two studies have investigated the effect of chia seeds in AD animal models so far, finding either no or detrimental effects on cognitive performance and on Aβ levels^26,27^. However, these studies were based on models that might not ideally mimic alterations of glucose metabolism occurring in AD, as present in genetically induced AD models.

This study further investigates the role of glucose metabolism in AD by the induction of metabolic stress in an AD mouse model. Based on the assumption that impairments in cerebral glucose metabolism represent an underlying mechanism of AD pathology, we hypothesize that (1) the AD mouse model displays disturbed glucose homeostasis and that (2) metabolic stress further aggravates cognitive symptoms and AD histopathology. Additionally, this study examines the therapeutic potential of chia seeds as a dietary supplement in AD. Assuming that chia seeds are able to improve glucose homeostasis, we hypothesize that (3) chia seed supplementation reverses or attenuates the effects of metabolic stress, thus ameliorating AD pathology and that (4) chia seed supplementation also leads to an improvement of AD pathology in mice that are not metabolically stressed due to their amelioration of underlying metabolic impairments.

## Materials and methods

### Animals

**Figure 1:**
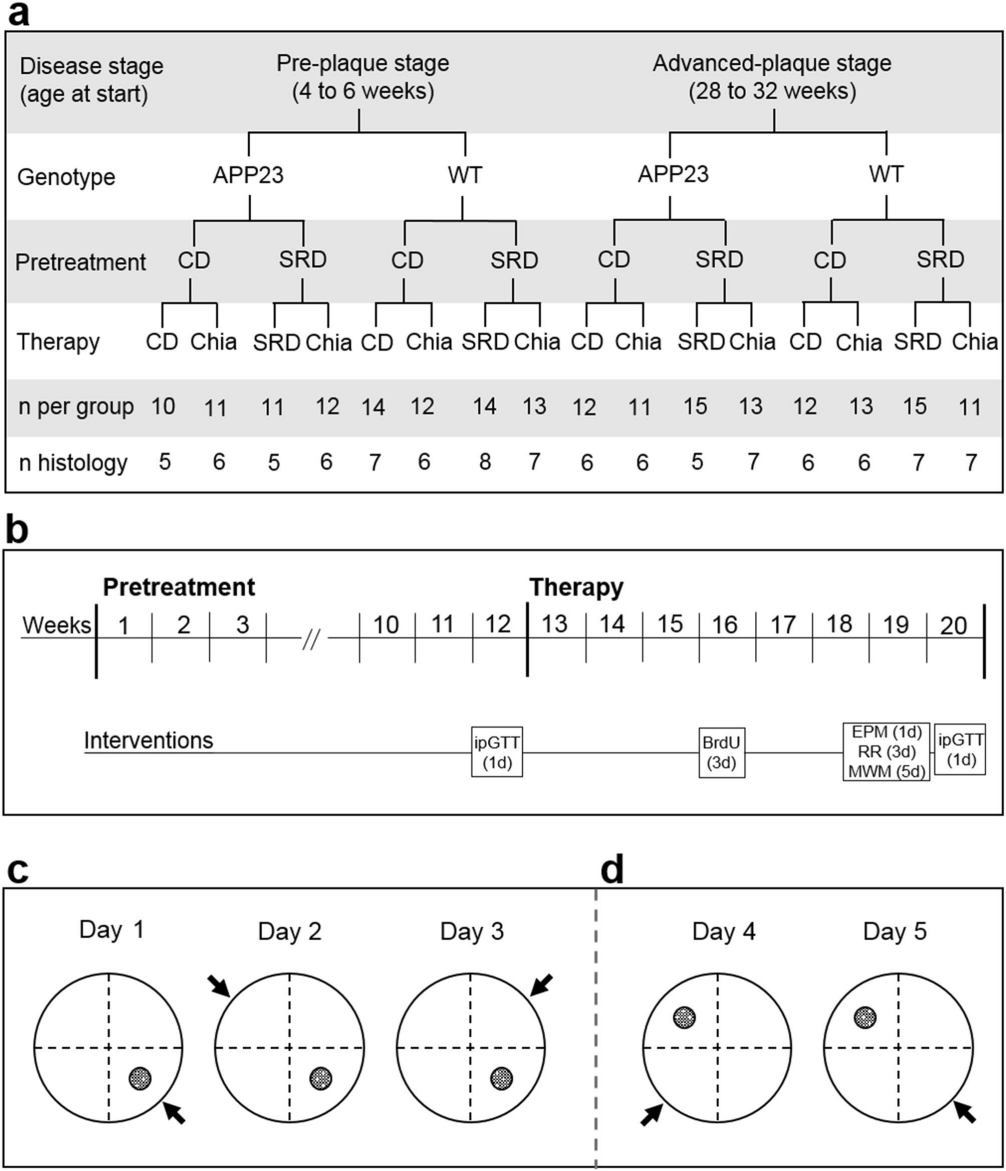
Study design (**a**,**b**) and reversal learning paradigm of the MWM (**c**,**d**). (**a**) The study was conducted in pre-plaque stage transgenic mice (PRE APP23 mice; 4–6 weeks old at start) and advanced-plaque stage transgenic mice (ADV APP23 mice; 28–32 weeks old at start) and age-matched healthy littermates (WT) as controls. Mice received either sucrose-rich diet (SRD) or control diet (CD) as a pretreatment. After pretreatment, mice underwent a therapy of either chia seed supplementation (CD + Chia or SRD + Chia) or of their respective diet from pretreatment (CD + CD or SRD + SRD) as a control. The last two rows display the group sizes for all measurements performed on the living animal and with the animals’ blood or for histological analyses of brain tissue, respectively. (**b**) Mice received pretreatment for 12 weeks, followed by 8 weeks of therapy. Glucose tolerance was assessed with an intraperitoneal glucose tolerance test (ipGTT) on the last day of pretreatment (week 12) and of therapy (week 20). In week 16, mice received injections of 5-bromo-2′-deoxyuridine (BrdU) on 3 consecutive days. At the end of therapy, mice underwent behavioral testing, using the Elevated Plus Maze (EPM) on 1 day, the Rotarod Performance Test (RR) on 3 consecutive days, and the Morris Water Maze (MWM) on 5 consecutive days. Mice were given a break of 2 days after the last day of MWM, before the second ipGTT was conducted. After 20 weeks of experiment, mice were sacrificed the day after the second ipGTT at the age of 24–26 weeks (PRE group) and 48–52 weeks (ADV group), respectively. (**c**) Position of the hidden escape platform (filled circle) and release position of mice for all trials of the respective day (arrow) during 3 days of acquisition phase in the MWM. (**d**) Position of the hidden escape platform (filled circle) and release position of mice for all trials of the respective day (arrow) during 2 days of reversal learning phase in the MWM.

Animal experiments were approved by the local animal ethics committee (Landesamt für Gesundheit und Soziales, Berlin, Germany; G0074/16) and carried out in accordance with the European Communities Council Directive of 22 September 2010 (10/63/EU). APP23 mice overexpress human APP_751_ cDNA with the Swedish double mutation under the murine Thy-1 promotor with Aβ plaque deposition starting at 6 months of age^28^. Breeding pairs were obtained from Novartis Pharma, the colony was maintained on a C57BL/6J background, and genotype was confirmed by PCR (Primers: APP ct forward: 5′ GAA TTC CGA CAT GAC TCA GG 3′, APP ct reverse: 5′ GTT CTG CTG CTG CAT CTT CGA CA 3′). Two age groups of female mice were observed: 44 APP23 mice and 53 healthy littermates (WT) entered the experiment aged 4–6 weeks old, (pre-plaque stage, PRE), 51 APP23 and 51 WT mice entered the experiment aged 28–32 weeks old (advanced-plaque stage, ADV). Animals were group-housed in a temperature- and humidity-controlled room (21.5 ± 1.5 °C, 40–60%) on a 12-h light/dark cycle with unrestricted access to food and water. Animals were randomly assigned to the different dietary conditions as described below (Fig. 1a). The experimenter was blinded to age, genotype and group allocation. The body weight (BW) of mice was measured weekly. 50 mg/kg BW 5-bromo-2′-deoxyuridine (BrdU) dissolved in sodium chloride were injected intraperitoneally (i.p.) every 24 h on 3 consecutive days in week 16 for examination of AHN (Fig. 1b).

### Diets

Mice received a 12 weeks long pretreatment of either a sucrose-rich diet (SRD) to induce metabolic stress or a control diet (CD) (Fig. 1a,b). SRD provided 4.28 kcal/g, CD provided 4.15 kcal/g, and both diets were composed of 17% protein, 23% fat and 60% carbohydrates. After 12 weeks, each group was randomly further subdivided into two groups for additional 8 weeks (Fig. 1a,b); the therapy group receiving their respective diet supplemented with 286 g/kg diet milled chia seeds (Onset Worldwide LC, Frenchtown, NJ, USA) (SRD + Chia or CD + Chia), the control group continuing with their original diet (SRD or CD). The amount of chia seeds added to the diets was based on a study of Chicco et al*.*^23^ and modified according to their content of nutrients and energy of the CD and SRD. SRD + Chia and CD + Chia had the same nutritional composition and value as SRD and CD, respectively. The exact composition of all diets and the nutritional facts of chia seeds can be found in Supplemental Tables S1 and S2.

### Intraperitoneal glucose tolerance test

Glucose tolerance was measured on the last day of pretreatment and on the last day of therapy (Fig. 1b) with an intraperitoneal glucose tolerance test (ipGTT) after a 6 h morning fast. Blood was obtained from a small incision of the distal tail vein after applying lidocaine/prilocaine ointment. Blood glucose was measured before (0 min) and 15, 30, 60, and 120 min after i.p. injection of 2 mg/g BW glucose solution. Glucose tolerance is expressed as area under the curve (AUC) in (mg/dl) × min. Higher blood glucose levels over a prolonged period of time result in a higher AUC, indicating a lower glucose tolerance.

### Behavioral testing of anxiety, motor coordination, and cognition

All behavioral tests were conducted during the last 2 weeks of therapy (Fig. 1b). The Elevated Plus Maze (EPM) measures anxiety-related behavior. Each mouse was placed in the center region facing a closed arm and had a single trial of 5 min. The software TSE VideoMot 3D Classic Version 8.02 automatically recorded the percentaged time mice spent in the open arms, the number of entries into the open arms and the latency of the first entry into an open arm.

The Rotarod Performance Test measures motor coordination and fatigue resistance and was conducted as reported previously^29^ to preclude limitations in motility that might negatively affect the Morris Water Maze (MWM) performance. Briefly, mice had a maximum running time of 5 min per trial with gradually accelerating rotation speeds from 4 to 40 rpm. The time mice balanced on the rod was measured by the software TSE-ROD Version 4.0. 2 days prior to the test day, mice were habituated to the procedure, performing 4 trials per day with an inter-trial-interval of 15–30 min. On the test day, the animals went through 3 equally conducted test trials. The mean of 3 trials was calculated.

In the MWM, spatial learning is assessed during the acquisition phase and cognitive flexibility is examined during a reversal learning phase. The MWM task was performed as previously described^29^. Briefly, the circular pool was filled with opaque water (22 ± 2 °C) and virtually divided into four quadrants with a circular escape platform submerged 1 cm below the water surface in the center of one quadrant (Fig. 1c,d). A different visual cue was placed at the wall above each quadrant. During the acquisition phase (day 1–3), the platform location remained the same (Fig. 1c), whereas it was shifted to the opposite quadrant during the reversal learning phase (day 4–5) (Fig. 1d). Each day consisted of 6 trials with an inter-trial-interval of 30–45 min. The starting position remained the same over these 6 trials, but was alternated each day (Fig. 1c,d, arrow). A trial was finished either with reaching the platform or after 2 min. If the mouse was unable to detect the platform, it was guided to its position after 2 min had elapsed. Each mouse was given 10 s to memorize the platform position. The software TSE VideoMot 2 Version 5.68 was used to automatically track the swim paths of mice, measuring a.o. the total distance covered to the escape platform. Swim trajectories were used to generate spatial presence probability maps using Matlab R2011b as described in the legend of Fig. 3.

### Perfusion and tissue preparation

Animals were deeply anesthetized after a 6 h morning fast via i.p. injection of 300 mg/kg BW ketamine hydrochloride and 30 mg/kg BW xylazine hydrochloride. Laparotomy and a final blood withdrawal from the *Inferior Vena Cava* were performed. Blood was mixed with 10 µl heparin per 500 µl and immediately centrifuged at 4 °C and 13,000 rpm for 3 min. Plasma was collected and instantly deep-frozen. Thoracotomy and transcardial perfusion using 40 ml of 0.1 M phosphate buffered saline (PBS) were performed. In half of each experimental group, the hippocampus was directly dissected from the fresh brain and deep-frozen in liquid nitrogen for analysis of inflammatory cytokine levels. In the other half, a second perfusion with 40 ml of 4% paraformaldehyde (PFA) in PBS was performed, before brains were removed, post-fixated overnight in 4% PFA in PBS at 4 °C, dehydrated for 48 h in 30% aqueous sucrose solution at 4 °C, and subsequently deep-frozen. Frozen coronal sections of 40 μm thickness were prepared for histological analyses with a cryostat.

### Histological analysis of plaque load, microglia, and adult hippocampal neurogenesis

Aβ plaque load was determined using the fluorescent pentameric oligothiophene (pFTAA) and only assessed in APP23 mice. A one-in-twelve series of brain slices containing the hippocampus was incubated for 30 min with 20 µg/ml pFTAA and counterstained with 4′,6-diamidino-2-phenylindole (DAPI). The percentaged area covered with Aβ plaques in both hippocampi and cortices was calculated automatically via the “Analyze Particles” tool of ImageJ in 6 sections per animal.

Brain slices containing the hippocampus were used for quantification of microglia and macrophages by staining against the ionized calcium-binding adapter molecule 1 (Iba1) in a one-in-twelve series. Sections of a one-in-six series were stained to assess AHN by quantification of survival of newborn cells, marked by the incorporation of BrdU, and by quantification of immature neurons, marked by the expression of doublecortin (DCX), as previously described^30^. Brain slices were pretreated with 0.6% H_2_O_2_. For BrdU, additional pretreatment with 2 M HCl was conducted. Subsequently, slices were incubated overnight at 4 °C with the primary antibody: polyclonal rabbit anti-Iba1 (Fujifilm Wako Chemicals Europe, 1:500), monoclonal rat anti-BrdU (Biozol, 1:500), or polyclonal guinea pig anti-DCX (Merck Millipore, 1:1000). The next day, the tissue was incubated with the secondary antibody for 2 h at room temperature: Biotin-SP-conjugated goat anti-rabbit, donkey anti-rat, or goat anti-guinea pig (each dianova, 1:250). This was followed by incubation with streptavidin peroxidase complex and the reaction was visualized by applying diaminobenzidine-nickel staining. The labeled microglia and macrophages in both hippocampi of 6 sections per animal were stereologically extrapolated using the “Optical Fractionator Probe” of MBF Bioscience Stereo Investigator with a grid size of 500 × 500 µm and a counting frame of 75 × 75 µm. The labeled BrdU^+^ and DCX^+^ cells in the granule cell layer and subgranular zone of both dentate gyri (DG) of 6 sections per animal were counted manually.

### Analysis of inflammatory cytokine levels in hippocampal tissue

A single hippocampus of 5 animals per group was suspended in radioimmunoprecipitation assay (RIPA) buffer mixed with protease inhibitor (100 µl buffer mix per 10 mg tissue) and mechanically minced. After 30 min of incubation on ice, samples were centrifuged at 13,000 rpm for 15 min at 4 °C and the supernatant was collected. Total protein concentration of samples was measured using the Pierce™ BCA Protein Assay Kit (Thermo Scientific) as described in the handbook. Samples were diluted 1:10 with PBS and a final volume of 10 µl was analyzed in duplicates. Concentrations of interferon-γ (IFN-γ), keratinocyte chemoattractant/human growth-regulated oncogene (KC/GRO), tumor necrosis factor-α (TNF-α) and interleukins (IL) IL-1β, IL-2, IL-4, IL-5, IL-6, IL-10, IL-12p70 were measured using the V-PLEX Plus Proinflammatory Panel1 Mouse Kit (Meso Scale Discovery) as described in the handbook. Duplicates of 50 µl undiluted samples were analyzed. Cytokine concentrations were normalized for total protein concentration. Measured levels of IL-10 were under detection range of the kit and thus excluded from the analysis.

### Quantification of fasting insulin and corticosterone levels in blood plasma

Blood plasma was analyzed using enzyme-linked immunosorbent assay (ELISA). Mouse Insulin ELISA kit (Mercodia) was used as described in the handbook to quantify fasting insulin levels. Duplicates of 5 µl undiluted plasma samples per animal were analyzed. Corticosterone ELISA kit (for human, rat and mouse, IBL International) was used as described in the handbook to analyze fasting corticosterone levels. For each animal, plasma samples were diluted 1:5 with the included “Standard 0” to a final volume of 20 µl and analyzed in duplicates.

### Statistical analysis

All data sets were analyzed with IBM SPSS Statistics 25 to verify, whether the data meet the assumptions for parametric testing. The Shapiro–Wilk test was performed to check for normal distribution and the Levene’s test was conducted to check for equal variances. In all data sets, one or more assumptions for parametric testing were violated, therefore nonparametric tests were chosen. Data sets were analyzed with R Version 3.6.3 using the packages nparcomp and nparLD as described elsewhere^31,32^. The function mctp from the package nparcomp was used to perform nonparametric multiple contrast test (type Tukey) between groups. The function f2.ld.f1 from the package nparLD was used to perform nonparametric ANOVA-type statistics of data sets that include repeated measures. The effects of the two independent factors (f2) genotype (g) and diet (d) and the longitudinal factor (f1) time (t) on the respective outcome parameters were analyzed. For further analyses, nparcomp was used to examine differences between groups at crucial time points within longitudinal data. The Rotarod performance was correlated with BW using Spearman’s rank correlation. A p-value ≤ 0.05 was considered significant. Results were plotted using GraphPad Prism 8.4.2.

## Results

### Chia seeds and SRD positively affect spatial learning impairments in APP23 mice

**Figure 2:**
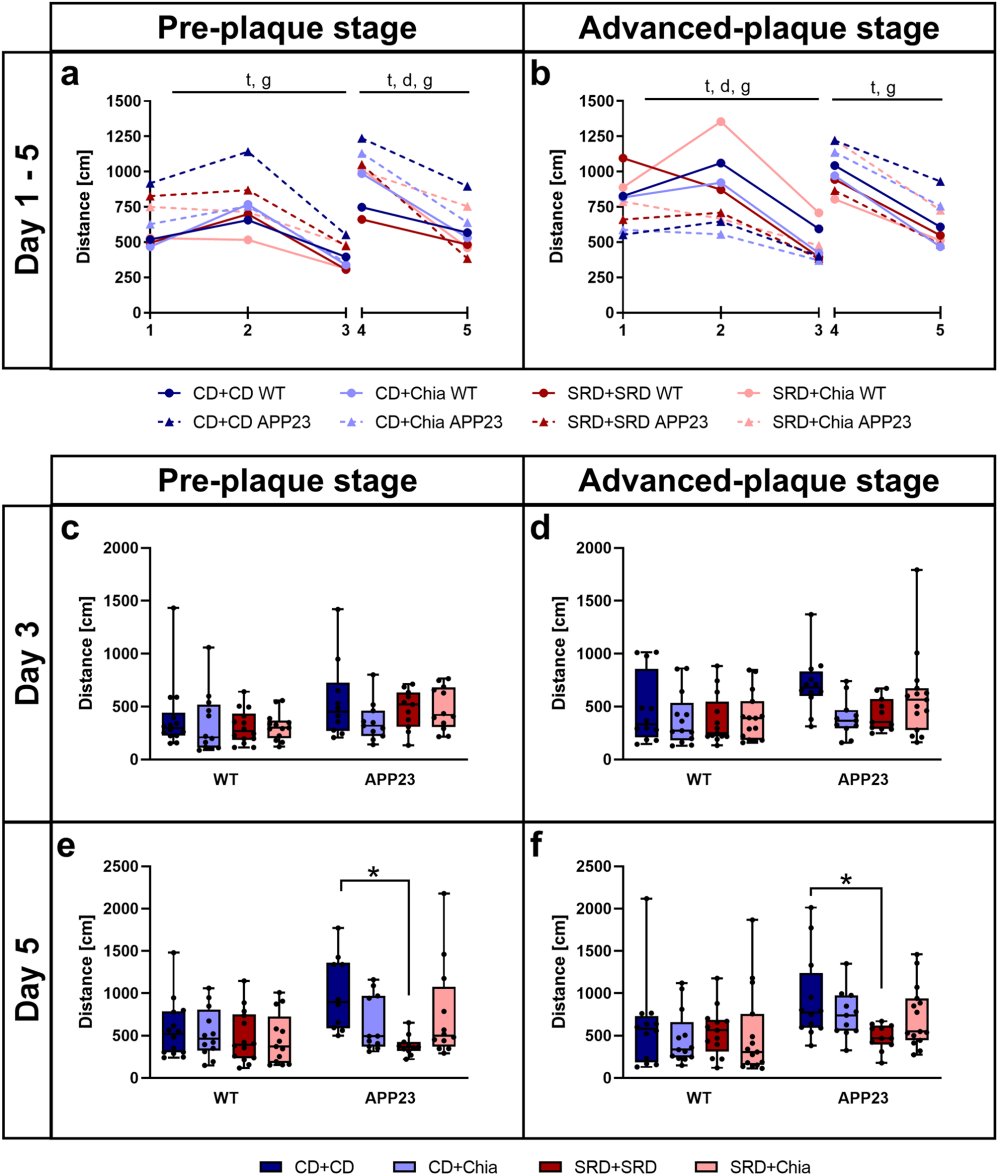
APP23 mice show impaired spatial learning, which is moderately improved by chia seeds, especially with age. APP23 mice show deteriorated reversal learning, which is improved by SRD. (**a**,**b**) Mean distance per day covered by PRE (**a**) and ADV mice (**b**) during acquisition phase (day 1–3) and reversal learning phase (day 4–5) in the MWM. Letters indicate significant factors (t = time, d = diet, g = genotype, combination of letters = interaction of two or three factors) regardless of significance level (p < 0.05), according to nonparametric repeated measures ANOVA-type test statistic. (**c**–**f**) Mean distance covered in the MWM by PRE (**c**, **e**) and ADV mice (**d**, **f**) on day 3, the last day of the acquisition phase, and day 5, the last day of the reversal learning phase, respectively. Each box represents the 25th to 75th percentile, the line represents the median, whiskers reach from minimum to maximum. An asterisk indicates significant differences between groups regardless of significance level (p < 0.05), according to nonparametric multiple contrast Tukey-type test. *WT* wild type control, *APP23* transgenic mouse model, *PRE* pre-plaque stage, *ADV* advanced-plaque stage, *CD* control, *SRD* sucrose-rich, *Chia* chia seed supplementation.

**Figure 3:**
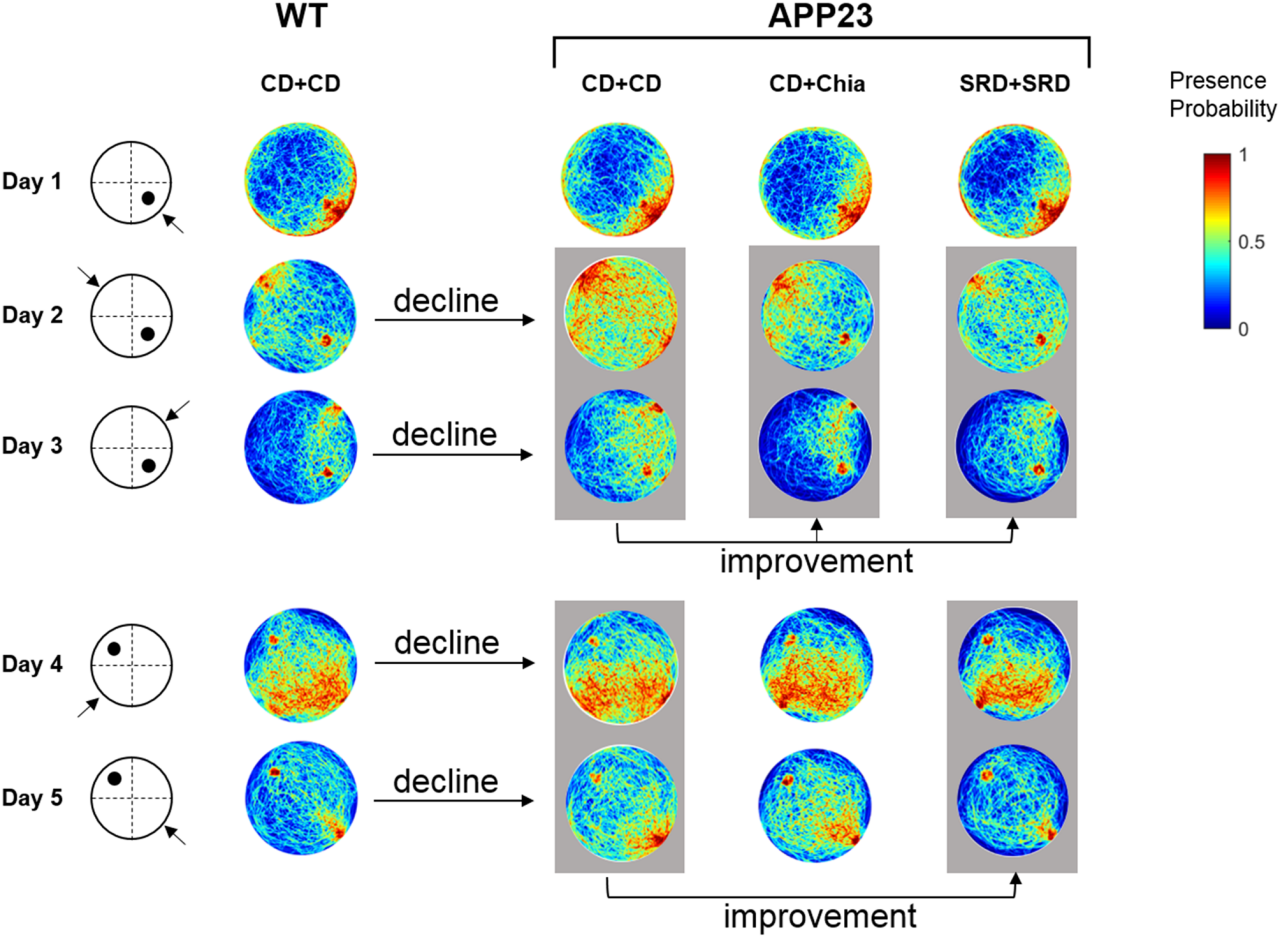
Visual representation of the effect of chia seeds and SRD during acquisition and reversal learning phase by exemplary pseudocolor coded heatmaps showing presence probabilities of ADV mice in the MWM. Reddish tones denote high presence probabilities and bluish tones denote low presence probabilities. The left panel indicates the day, the platform position (black dot) and the starting position of mice (arrow). The first row of heatmaps shows the presence probabilities of WT mice receiving CD + CD. The second row of heatmaps represents the presence probabilities of ADV APP23 mice receiving CD + CD, showing notably less directed swimming trajectories. The third row of heatmaps represents the presence probabilities of ADV APP23 mice receiving CD + Chia. Day 2 and 3 (marked by gray box) show more targeted swimming trajectories, compared to APP23 mice on CD + CD. The last row of heatmaps represents the presence probabilities of ADV APP23 mice receiving SRD + SRD. Day 2 and 3, as well as day 4 and 5 (marked by gray boxes), show more targeted swimming trajectories compared to APP23 mice receiving CD + CD. Heatmaps were generated with Matlab R2011b as follows. First, for each mouse swim paths of all 6 trials per day were projected to planar space as binary images (swim trajectory was set to value 1, non-swim path was set to value 0). Second, a single swim path per mouse and day was obtained using the mathematical union operation with values set to 1, if the animal traversed this position in at least 1 out of 6 trials. Third, probabilistic maps were created based on the binary images of all mice in one group on one day, such that the probability of 1 was assigned to a pixel that was crossed by every mouse in the respective group on the respective day. Conversely, a probability of 0 was allocated to a pixel that was never traversed by any mouse in the respective group on the respective day. *WT* wild type control, *APP23* transgenic mouse model, *PRE* pre-plaque stage, *ADV* advanced-plaque stage, *CD* control, *SRD* sucrose-rich, *Chia* chia seed supplementation.

The daily MWM performance of mice is expressed as the mean distance covered to the escape platform of all trials (Fig. 2). Figure 3 shows exemplary heatmaps of presence probabilities. Complete heatmaps can be found in Supplemental Figure S3. Mice of both the PRE and the ADV group successfully learned the platform position during the acquisition phase, represented by a significant time-dependent reduction of the distance covered to the platform (PRE: F(1.682,78.596) = 38.286, p < 0.001; ADV: F(1.684,84.952) = 32.354, p < 0.001) (Fig. 2a,b). However, PRE and ADV APP23 mice performed inferior to age-matched WT mice during the acquisition phase (PRE: F(1,78.596) = 21.148, p < 0.001; ADV: F(1,84.952) = 25.685, p < 0.001), as shown by up to 46% longer distances (Fig. 2a,b). Only in the ADV group, diet significantly improved the performance of mice (F(2.838,84.952) = 3.275, p = 0.022), as seen by a 16% distance reduction each due to CD + Chia and SRD + Chia and unexpectedly also a 25% distance reduction due to SRD + SRD (Fig. 2b). In summary, PRE and ADV mice successfully learned the platform position during acquisition phase, but APP23 mice covered longer distances and thus showed weaker spatial learning capabilities than WT mice.

At the last day of acquisition (day 3), no statistically significant differences between groups were detectable, indicating that all mice learned the platform position equally well. Nevertheless, we want to point out some notable differences. Both PRE and ADV APP23 mice had more difficulties in detecting the escape platform than age-matched WT mice, reflected by up to 54% longer distances (Fig. 2c,d). Interestingly, CD + Chia improved the performance of both PRE and ADV APP23 mice, as represented by an almost equal distance covered to the platform compared to age-matched WT mice (Fig. 2c,d). In ADV APP23 mice, CD + Chia even reduced the covered distance by 36% compared to CD + CD (p = 0.078) (Fig. 2d). Unexpectedly, also SRD + SRD improved the performance of ADV APP23 mice by reducing the covered distance by 14% compared to CD + CD (p = 0.068) (Fig. 2d). Both, the effect of chia supplementation and SRD, were also apparent as more targeted swim patterns in the heatmaps (Fig. 3, Supplemental Figure S3). In summary, both chia supplementation as well as SRD positively affected spatial learning in APP23 mice, especially with age.

### SRD improves impaired cognitive flexibility in APP23 mice

During the reversal learning phase, mice of both the PRE and the ADV group successfully learned the new platform position, reflected by a significant time-dependent reduction of the distance covered to the platform (PRE: F(1,82.512) = 55.473, p < 0.001; ADV: F(1,89.701) = 106.916, p < 0.001) (Fig. 2a, b). Consistently, PRE and ADV APP23 mice performed inferior to age-matched WT mice also during the reversal learning phase (PRE: F(1,82.512) = 12.035, p < 0.001; ADV: F(1,89.701) = 10.169, p < 0.001), as shown by up to 30% longer distances (Fig. 2a,b). In both the PRE and ADV group, diet improved the performance of mice (PRE: F(2.924,82.512) = 3.760, p = 0.010; ADV: F(2.968,89.701) = 2.558, p = 0.053), although this missed statistical significance in the ADV group. The effect of diet was represented by a distance reduction of up to 25% due to SRD + SRD (Fig. 2a,b). In summary, all mice successfully re-learned the platform position during the reversal learning phase, with APP23 mice again covering longer distances and thus showing less cognitive flexibility.

At the last day of reversal learning (day 5), SRD + SRD significantly improved the performance of both PRE and ADV APP23 mice compared to CD + CD, represented by a distance reduction of 57% (p < 0.001) and 48% (p = 0.048), respectively (Fig. 2e,f). The same effect was visible as more targeted swim patterns in the heatmaps (Fig. 3, Supplemental Figure S3). In summary, SRD improved cognitive flexibility in both PRE and ADV APP23 mice, whereas chia seeds had no effect on reversal learning.

### Chia seeds positively influence glucose tolerance with age

**Figure 4:**
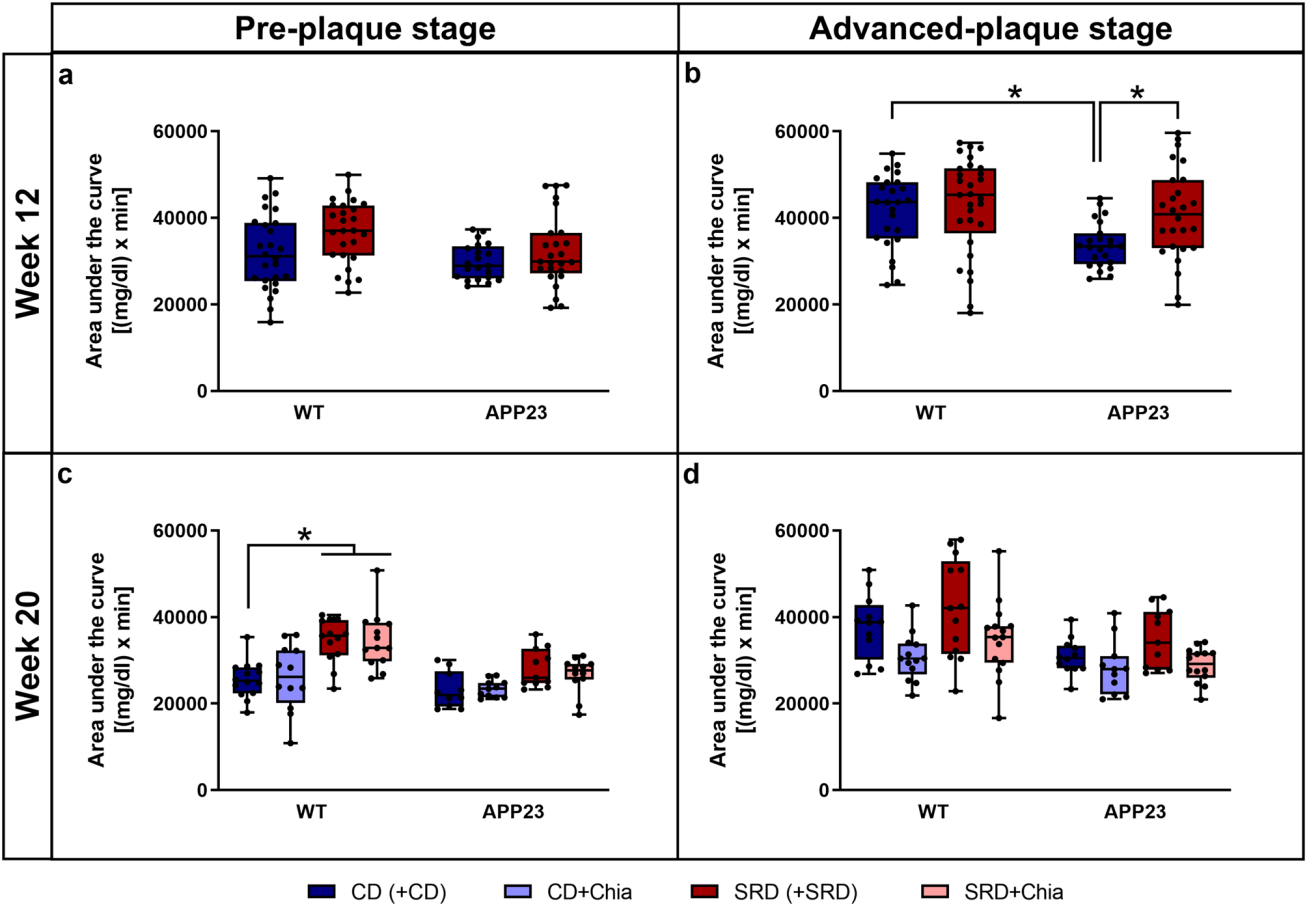
APP23 mice show a better glucose tolerance than WT mice, but ADV APP23 mice are more vulnerable to SRD-induced glucose intolerance. Glucose tolerance can be moderately improved by chia seeds in ADV APP23 and age-matched WT mice. (**a**,**b**) Glucose tolerance of PRE (**a**) and ADV mice (**b**) represented by area under the curve in (mg/dl) × min in week 12 at the end of pretreatment. (**c**,**d**) Glucose tolerance of PRE (**c**) and ADV mice (**d**) represented by area under the curve in (mg/dl) × min in week 20 at the end of therapy. Each box represents the 25th to 75th percentile, the line represents the median, whiskers reach from minimum to maximum. An asterisk indicates significant differences between groups regardless of significance level (p < 0.05), according to nonparametric multiple contrast Tukey-type test. *WT* wild type control, *APP23* transgenic mouse model, *PRE* pre-plaque stage, *ADV* advanced-plaque stage, *CD* control, *SRD* sucrose-rich, *Chia* chia seed supplementation.

In the PRE group, no significant differences in the AUC of the ipGTT were detectable after 12 weeks of pretreatment, indicating that SRD did not induce glucose intolerance in any genotype (Fig. 4a). However, after additional 8 weeks of therapy, glucose tolerance of WT mice of the PRE group was impaired due to SRD, as shown by an up to 35% increased AUC (SRD + SRD: p = 0.027; SRD + Chia: p = 0.008) (Fig. 4c).

In the ADV group, 12 weeks of pretreatment resulted in impaired glucose tolerance in APP23 mice, indicated by a 21% increase in the AUC of APP23 mice due to SRD (p = 0.015) (Fig. 4b). In contrast, glucose tolerance of WT mice was not impaired after 12 weeks of SRD (Fig. 4b). Interestingly, not metabolically stressed APP23 mice showed significantly superior glucose tolerance compared to WT mice, as shown by a 19% smaller AUC (p = 0.001) (Fig. 4b). In the ADV group, we could not detect significant differences in the AUC after 8 weeks of therapy. However, we want to point out that both CD + Chia and SRD + Chia ameliorated glucose tolerance, indicated by a 13% reduction of AUC in APP23 mice and a 18% reduction of AUC in WT mice (Fig. 4d). To sum up, although ADV APP23 mice showed an overall better glucose tolerance than age-matched WT mice, they were more prone to SRD-induced glucose intolerance. Chia seeds mildly ameliorated glucose tolerance in mice of both genotypes in the ADV group.

### Old APP23 mice show mild SRD-induced insulin insensitivity and generally elevated corticosterone levels, which are positively affected by chia seeds

**Figure 5:**
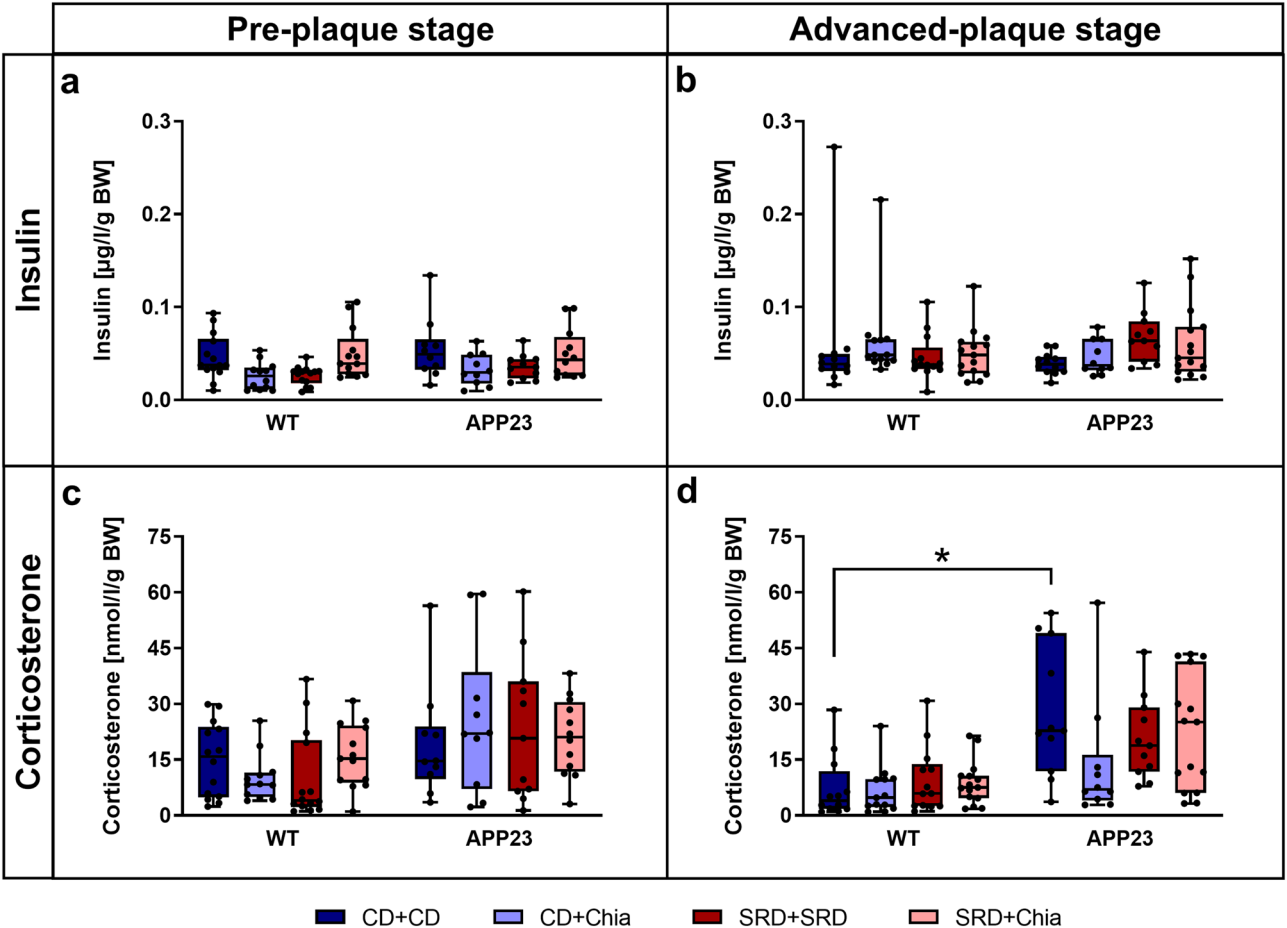
ADV APP23 mice show slight SRD-induced insulin insensitivity and generally increased corticosterone levels compared to age-matched WT mice, the latter being moderately improved by chia seeds. (**a**,**b**) Fasting plasma insulin levels adjusted for BW [µg/l/g] of PRE (**a**) and ADV mice (**b**). (**c**,**d**) Fasting plasma corticosterone levels adjusted for BW [nmol/l/g] of PRE (**c**) and ADV mice (**d**). Each box represents the 25th to 75th percentile, the line represents the median, whiskers reach from minimum to maximum. An asterisk indicates significant differences between groups regardless of significance level (p < 0.05), according to nonparametric multiple contrast Tukey-type test. *WT* wild type control, *APP23* transgenic mouse model, *PRE* pre-plaque stage, *ADV* advanced-plaque stage, *CD* control, *SRD* sucrose-rich, *Chia* chia seed supplementation, *BW* body weight.

Fasting insulin and corticosterone levels are displayed adjusted for BW (Fig. 5), since both hormones are linked to the regulation of BW. Insulin levels did not significantly differ between mice of the PRE group (Fig. 5a). Although insulin levels did not significantly differ in the ADV group either, we want to point out that SRD + SRD increased insulin levels by on average 74% in ADV APP23 mice compared to CD + CD (p = 0.079) (Fig. 5b). In summary, we observed that ADV APP23 mice might show slight insulin insensitivity induced by SRD. Insulin levels were not affected by chia seeds.

Corticosterone levels did not significantly differ between mice of the PRE group (Fig. 5c). Generally, ADV APP23 mice displayed on average 2.5 times higher corticosterone levels than age-matched WT mice (Fig. 5d). Interestingly, chia seeds reduced corticosterone levels by 51% in ADV APP23 mice compared to CD + CD. However, this difference was not statistically significant (p = 0.349) (Fig. 5d). In summary, ADV APP23 mice showed elevated corticosterone levels compared to age-matched WT mice. Nevertheless, chia seeds exerted a mild positive effect on corticosterone levels in ADV APP23 mice.

### Chia seeds reverse SRD-induced elevation of pro-inflammatory cytokine levels, whereas Aβ plaque load and microglia abundance are not affected by diet

**Figure 6:**
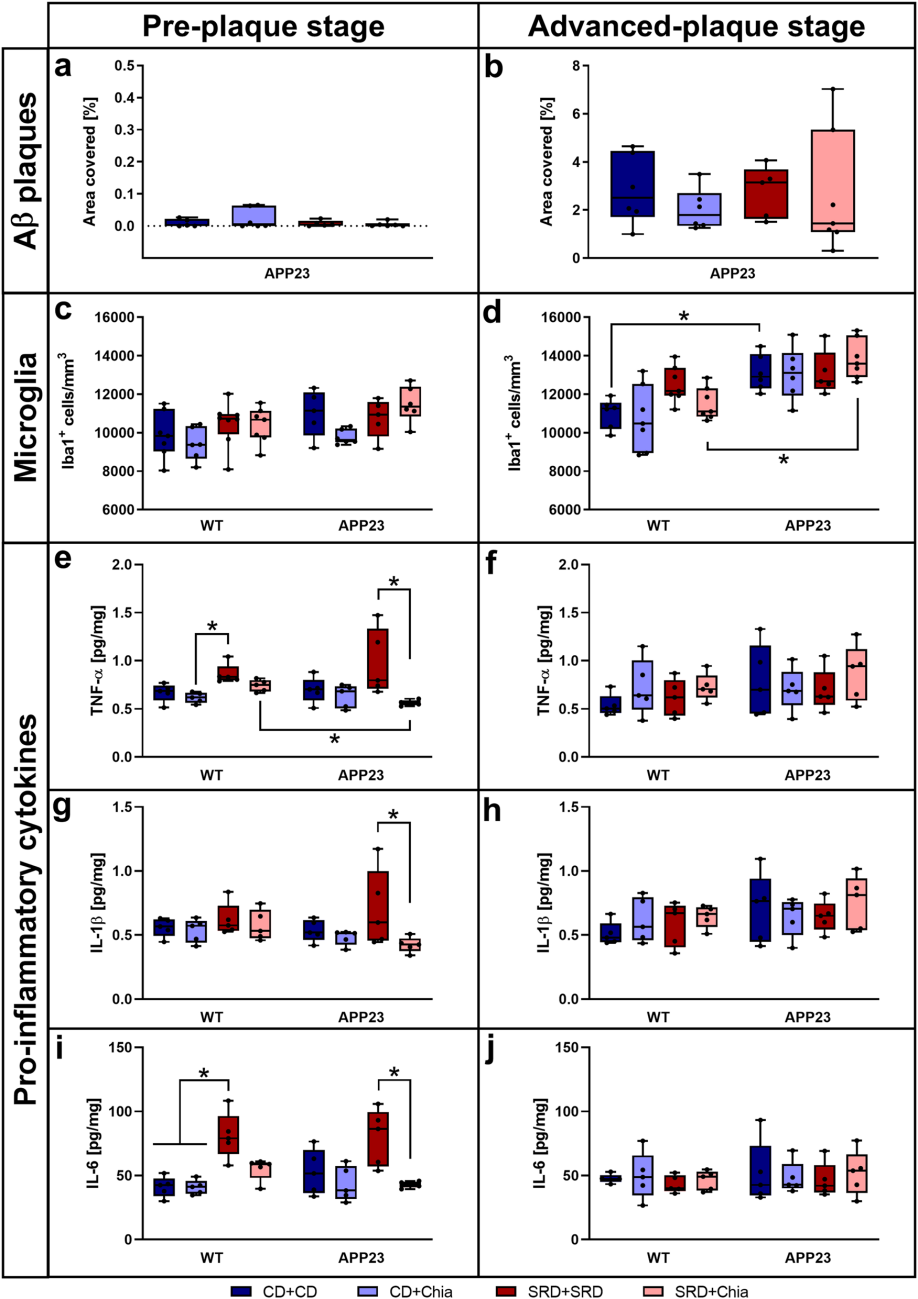
ADV APP23 mice show increased Aβ plaque load and elevated numbers of microglia in the hippocampus. Pro-inflammatory cytokine levels are increased by SRD but only in the PRE group. (**a**,**b**) Aβ plaque load represented by the percentage of area covered with pFTAA-positive plaques in the hippocampus and cortex of PRE (**a**) and ADV mice (**b**). (**c**,**d**) Number of microglia/macrophages expressing Iba1^+^ per mm^3^ in the hippocampus of PRE (**c**) and ADV mice (**d**). (**e**,**f**) TNF-α levels [pg/mg] in the hippocampus of PRE (**e**) and ADV (**f**) mice. (**g**,**h**) IL-1β levels [pg/mg] in the hippocampus of PRE (**g**) and ADV (**h**) mice. (**i**,**j**) IL-6 levels [pg/mg] in the hippocampus of PRE (**i**) and ADV (**j**) mice. Each box represents the 25th to 75th percentile, the line represents the median, whiskers reach from minimum to maximum. An asterisk indicates significant differences between groups regardless of significance level (p < 0.05), according to nonparametric multiple contrast Tukey-type test. *WT* wild type control, *APP23* transgenic mouse model, *PRE* pre-plaque stage, *ADV* advanced-plaque stage, *CD* control, *SRD* sucrose-rich, *Chia* chia seed supplementation, *Iba1* allograft inflammatory factor 1, *TNF-α* tumor necrosis factor-α, *IL* interleukin.

PRE APP23 mice only showed very few and very small plaques in hippocampus and cortex and there were no differences in Aβ plaque load between the dietary groups (Fig. 6a). Aβ plaque load in ADV APP23 mice was more than 250 times higher compared to PRE APP23 mice (on average 2.57% of hippocampus and cortex covered with plaques) but also showed no differences between dietary groups (Fig. 6b). No statistically significant differences in the number of hippocampal microglia were detectable in mice of the PRE group (Fig. 6c). Generally, ADV APP23 mice showed about 20% increased amounts of hippocampal microglia compared to age-matched WT mice (within CD + CD: p = 0.025; within SRD + Chia: p = 0.020) (Fig. 6d). Nevertheless, diet did not affect the number of hippocampal microglia. Representative images of pFTAA- and Iba1-stainings are presented in Supplemental Figure S4.

The analysis of pro-inflammatory cytokines was focused on TNF-α, IL-1β, and IL-6 as early players in the switch of microglia from an anti-inflammatory to a pro-inflammatory phenotype. However, results of the remaining measured pro-inflammatory cytokines were very similar and are presented in Supplemental Figure S5. In the PRE group, hippocampal levels of pro-inflammatory cytokines were similar between APP23 and WT mice (Fig. 6e,g,i). TNF-α levels were significantly increased due to SRD by 40% in WT mice (CD + Chia vs. SRD + SRD, p = 0.008) and by 73% in APP23 mice (SRD + SRD vs. SRD + Chia, p = 0.049) (Fig. 6e). Similarly, hippocampal IL-1β levels were significantly increased due to SRD by 65% in APP23 mice (SRD + SRD vs. SRD + Chia, p = 0.040) (Fig. 6g). Consistently, IL-6 levels were significantly elevated due to SRD by 98% in WT mice (CD + CD/CD + Chia vs. SRD + SRD, p = 0.048 and p = 0.027) and by 86% in APP23 mice (SRD + SRD vs. SRD + Chia, p = 0.047) (Fig. 6i). Interestingly, all pro-inflammatory cytokine levels were normalized due to chia supplementation of SRD in the PRE group (Fig. 6e,g,i). In contrast, there were no significant differences detectable in the ADV group (Fig. 6f,h,j). In summary, both Aβ plaque load and microglia abundance were increased in ADV APP23 mice but unaffected by diet. In contrast, pro-inflammatory cytokine levels were similar between genotypes but elevated by SRD in the PRE group but not in the ADV group. This effect was reversed by chia seeds.

Cell survival of newborn cells and the number of immature neurons together with representative images are shown in Supplemental Figure S6. In summary, AHN was not altered in APP23 mice and unaffected by diet.

### Reduced body weight in APP23 mice tends to be increased by chia seeds

The development of BW over time is shown in Supplemental Figure S7. In summary, APP23 mice weighed significantly less than WT mice with the difference becoming more pronounced with age (up to 15% difference in BW in the PRE group and up to 22% in the ADV group) (PRE: F(1,71.940) = 71.498, p < 0.001; ADV: F(1,64.711) = 136.102, p < 0.001). Chia seeds moderately increased BW by about 10% in the PRE group and about 9% in the ADV group (PRE: F(2.823,71.940) = 8.763, p < 0.001; ADV: F(2.873,64.711) = 3.692, p = 0.012).

### Anxiety-related behavior is neither altered by genotype nor by diet

Supplemental Figure S8 shows the analysis of animals’ behavior in the EPM. In summary, anxiety-related behavior did not differ between APP23 and WT mice, neither in the PRE nor in the ADV group. It was also not affected by any diet.

### Motor coordination negatively correlates with body weight

Motor coordination is displayed as the time until mice fell off the accelerating rotating rod in Supplemental Figure S9. In summary, there were no relevant differences between any of the groups. Nevertheless, time on the rod negatively correlated with BW of mice (PRE: r = -0.4212, p < 0.001; ADV: r = − 0.4102, p < 0.001), i.e. mice weighing more spent less time on the rod. Since APP23 mice weighed significantly less than WT mice and hence showed better motor coordination, we conclude that the observed inferior performance of APP23 mice in the MWM is truly related to cognitive impairment and not to motoric deficits.

## Discussion

This study was conducted to examine the effects of diet-induced metabolic stress as well as the therapeutic potential of chia seeds in AD pathology.

PRE and ADV APP23 mice showed impaired spatial learning and cognitive flexibility. Cognitive deficits of APP23 mice have been demonstrated before by us and others, in some studies even preceding the onset of Aβ plaque deposition^29,33^. The fact that cognitive performance was already affected in PRE APP23 mice, which scarcely displayed Aβ plaques, further supports the idea that Aβ plaques might not represent the neuropathologic correlate and exclusive underlying mechanism of cognitive decline in AD.

Diet differentially influenced both spatial learning and cognitive flexibility. We showed for the first time that chia seed supplementation mildly improved spatial learning especially in ADV APP23 mice. Our data suggest that this effect might be due to an accumulation of several aspects, although some of them on their own did not reach statistical significance. Firstly, chia seeds seemed to slightly improve glucose tolerance in ADV APP23 mice. It has been shown that impaired glucose tolerance correlates with cognitive decline and that the amelioration of glucose homeostasis positively affects cognitive performance^34,35^. Secondly, chia seeds led to a slight reduction of elevated corticosterone levels in ADV APP23 mice. Studies have shown that long-term elevated corticosterone levels induce cognitive deficits and that a reduction of corticosterone levels ameliorates cognitive impairment^36,37^. Thirdly, chia seeds reversed SRD-induced elevation of pro-inflammatory cytokine levels in the PRE group. Recent studies suggest that enhanced neuroinflammation might directly correlate with inferior cognitive perfomance^38^. Lastly, chia seeds are rich in PUFAs, which are further metabolized into EPA and DHA^18,20^. EPA and DHA have been shown to stimulate synaptogenesis, to act neuroprotective, to improve long-term potentiation and to ameliorate Aβ-induced cognitive deficits^39,40^. Thus, these minor contributions might add up to an improvement of spatial learning. Nevertheless, chia seeds failed to ameliorate impaired cognitive flexibility in APP23 mice. This seems controversial, since supplementation with DHA has been shown to improve reversal learning in healthy mice and reduction of corticosterone levels has been shown to correlate with improved cognitive flexibility in healthy rats^41,42^. However, reversal learning requires the suppression of the previously learnt platform position, cognitive flexibility to imprint the new position, as well as attention and motivation^43^. Therefore, a high degree of functional plasticity is needed, which is clearly impaired in AD^44,45^. Reduction of corticosterone levels or DHA supplementation might be adequate to improve cognitive flexibility in otherwise healthy animals. However, it appears to be insufficient in diseased animals used in the present study, potentially due to a lack of functional plasticity.

In contrast to our initial hypothesis, SRD improved both spatial learning and cognitive flexibility especially in ADV APP23 mice. Numerous studies have shown the detrimental effects of energy-rich diets on cognitive abilities, such as spatial and reversal learning^46,47^. However, most of these studies have compared energy-rich diets to normal lab chow, thus the observed effects might not primarily depend on sugar or fat itself but rather on increased calorie intake. The detrimental effect of high calorie intake on brain function and AHN has been shown before^48^. In the present study, we used isocaloric diets in order to ensure similar calorie intake in all groups. We hypothesize that the observed beneficial effect of SRD on cognition might be mediated by a fast access to energy supplied by short-chained carbohydrates. APP23 mice, which were—especially in the ADV group—substantially leaner compared to WT mice, potentially profited from this quickly accessible energy. In conclusion, rapidly accessible energy delivered by SRD might have provided mice with more capacities for cognitive tasks. A less physically challenging cognitive test, such as the Barnes Maze, might have been more suitable for this mouse model.

PRE and ADV APP23 mice treated with CD showed better glucose tolerance than WT mice. This contradicts our initial hypothesis of inferior glucose tolerance of APP23 mice. However, while cerebral glucose hypometabolism is reliably detectable very early in disease progression^49^, alterations of peripheral glucose metabolism do not always occur in relation with AD. Both human and animal studies have revealed controversial outcomes, in which peripheral glucose tolerance and insulin sensitivity in AD were either inferior, not altered or even superior compared to controls^50–56^. For example, intact glucose tolerance has been shown in male APP23 mice of different ages^56^ and in both male and female APP/PS1 mice^54^. On the one hand, these inconsistencies might result from different disease models, the influence of the estrous cycle in females and different protocols of the highly stress-sensitive glucose tolerance test. On the other hand, it has been shown that peripheral glycemic dysregulation in AD might be mediated by peripheral Aβ levels, which are very low in APP23 mice^10,56^. Thus, APP23 mice might not ideally mimic peripheral AD-induced metabolic alterations. Additionally, the significantly lower BW of APP23 mice might have accounted for better glucose tolerance compared to WT mice, since weight loss is a key intervention in the improvement of glucose tolerance^57^. Consistently with not being glucose intolerant per se, APP23 mice fed with CD did not show insulin resistance compared to WT mice.

We showed for the first time that ADV but not PRE APP23 mice displayed impaired glucose tolerance due to SRD. It has been shown that age drives an incline in glucose tolerance over time^58^. Our findings are in line with other studies showing that aged APP/PS1 mice are more prone to diet-induced glucose intolerance, when challenged with a high-fat diet^59^. These findings suggest that the presence of Aβ, potentially together with an age-related decrease of glucose tolerance per se, promotes diet-induced glucose intolerance. A possible mechanism might be interference of Aβ with hypothalamic insulin signaling. This has been shown to be crucial for the inhibition of further glucose production, thus leading to peripheral hyperglycemia^53,60^. In line with these findings, ADV APP23 mice showed a mildly enhanced vulnerability to SRD-induced insulin insensitivity.

Chia seeds improved glucose tolerance in both genotypes of the ADV group but not of the PRE group, although this difference was not statistically significant. This supports earlier findings, which have shown that chia seeds decrease diet-induced elevated plasma glucose level in rats^23,24^ and improve glycemic control in humans^61,62^. Since ageing itself drives glucose intolerance, as described above, it is plausible that the positive effect of chia seeds is more prominent in ADV mice than in PRE mice.

ADV but not PRE APP23 mice showed notably increased levels of corticosterone compared to WT mice, indicating a dysregulation of the HPA axis. The hippocampus plays a crucial role in HPA axis regulation^63^. It has been shown that intracerebroventricular injection of Aβ in healthy rats results in disturbed feedback of the HPA axis^64^. Considering the early affection of the hippocampus in AD, elevated GCs observed in AD patients might reflect an HPA axis dysregulation as a reaction to Aβ toxicity^65^. This matches our observation that PRE APP23 mice barely exhibited Aβ deposits and showed normal corticosterone levels, whereas ADV APP23 mice exhibited significant amounts of Aβ plaques and displayed increased corticosterone levels. Additionally, elevated levels of GCs have been shown to deteriorate insulin sensitivity and glucose tolerance^66^. Therefore, we suggest that Aβ-induced HPA axis dysregulation in ADV APP23 mice led to increased corticosterone levels, which in turn resulted in glucose intolerance and moderate insulin insensitivity, when mice were challenged with SRD. Due to the absence of Aβ deposits in PRE APP23 mice, corticosterone levels, glucose tolerance and insulin sensitivity were unaffected.

Aβ plaque load and the number of microglia were considerably increased in ADV APP23 mice compared to PRE APP23 mice and age-matched WT mice, respectively. These findings are consistent with previous descriptions of plaque pathology slowly starting at 6 months of age and of Aβ plaques being surrounded by activated microglia in APP23 mice^28,67^. However, pro-inflammatory cytokine levels were similar between APP23 and WT mice of both age groups. Thus, although more abundant in ADV APP23 mice, microglia do not seem to release higher levels of pro-inflammatory cytokines. This is in line with a longitudinal study in APP23 mice showing that neuroinflammation in the hippocampus first occurs at 20 months of age^68^. Furthermore, estrogen directly influences microglial responses. For example, estrogen supplementation in ovariectomized APP23 mice has been shown to reduce the expression of pro-inflammatory cytokines^69^. Thus, the low levels of pro-inflammatory cytokines despite elevated numbers of microglia might be due to an estrogen-mediated delay in the inflammatory response of microglia to Aβ plaques^69^. Pro-inflammatory cytokine levels were increased by SRD but only in the PRE group. The inflammatory response to SRD is in line with earlier studies that have demonstrated increased neuroinflammation due to excessive sugar intake^70^. It has also been shown that this response is age-dependent. Though, normally aged individuals are more prone to diet-induced neuroinflammation than young individuals^71^. Here, younger mice show a more pronounced inflammatory response to SRD. This might be due to elevated corticosterone levels in the ADV group, since GCs counteract the pro-inflammatory action of cytokines^72^. Nevertheless, corticosterone levels were only elevated in ADV APP23 mice, hence giving no explanation for the absence of an inflammatory response to SRD in aged WT mice, the reasons for which we can only speculate about. A possible explanation might lie in the composition of gut microbiota, which have been shown to influence neuroinflammation via the gut-brain axis and to be superior in WT mice compared to AD mouse models^73^. SRD-induced elevation of pro-inflammatory cytokine levels in the PRE group was entirely reversed by chia seed supplementation. We suggest that this anti-inflammatory effect might be mediated by omega-3 PUFAs, since it has been shown that EPA and DHA decrease the expression of pro-inflammatory factors^21^. The number of microglia and Aβ plaque load were not affected by diet. In contrast to our results, it has been shown that DHA supplementation is able to reduce Aβ deposition in two different AD mouse models overexpressing APP^74,75^. ALA and EPA instead increase Aβ production *in vitro*^75^. Thus, ALA and EPA might counteract the beneficial effects of DHA, which leads to unaltered Aβ burden in our study.

APP23 mice weighed significantly less than WT mice, the difference becoming more prominent with age, as already observed by others^76,77^. Weight loss also occurs in AD patients, the causative reasons remaining elusive^78^. We addressed this question in a different study. First analyses suggest an increased energy expenditure, respiratory exchange rate, and hyperactivity of APP23 mice (data not yet published). Chia seeds showed an overall tendency of inducing weight gain. Others observed increased food intake and subsequent weight gain in rats due to chia seed supplementation^24^. We also observed enthusiastic feeding on food pellets including chia seeds, potentially leading to weight gain. A human study instead reported moderate weight loss in overweight and obese patients due to chia seed supplementation^79^. These different findings might result from ad libitum feeding in animal studies, whereas calorie intake was restricted in the human study.

The genetic induction of Aβ deposition in the APP23 mouse model might limit the insights into the relationship of glucose metabolism and AD, since Aβ pathology in this case obviously represents the underlying mechanism. However, animal models accurately modeling sporadic late-onset AD still have to be developed. Although we intentionally chose female mice considering that AD is more prevalent in women, the estrous cycle potentially interferes with other hormone systems such as the HPA-axis. Hence, future studies should carefully monitor the estrous cycle to assess its impact on study outcomes. Another aspect that might limit the significance of chia seed supplementation in AD is the high dose used in this and other animal studies as such a high dose might not be applicable to a person’s diet. However, already lower doses of chia seeds successfully improved metabolic features in human studies, making a similar effect on cognition possible.

## Conclusion

We initially hypothesized that APP23 mice would display a disturbed peripheral glucose metabolism. Contrary, we show here that the peripheral glucose metabolism of APP23 mice is generally functioning. Nevertheless, we show for the first time an age-dependent increase in vulnerability to metabolic stress in APP23 mice. But in contrast to our hypothesis that metabolic stress would aggravate AD pathology in APP23 mice, cognitive performance as well as Aβ plaque load are not negatively influenced by SRD, despite a SRD-induced elevation of pro-inflammatory cytokine levels. This suggests that glucose hypometabolism might not be the underlying mechanism driving AD pathology in this AD mouse model. Furthermore, we hypothesized that chia seeds would attenuate AD pathology by improving metabolic parameters. Our data reveal a moderate therapeutic potential of chia seeds in alleviating spatial learning impairments by a mild amelioration of glucose tolerance, a slight reduction of corticosterone levels, and a reversal of SRD-induced elevation of pro-inflammatory cytokine levels.

## Supplementary information

Supplementary Information

## Data Availability

The datasets generated during and/or analysed during the current study are available from the corresponding author on reasonable request.
