# Supplementary material for: Chia seeds as a potential cognitive booster in the APP23 Alzheimer’s disease model
Source: Sci Rep. 2020 Oct 26;10:18215. doi: 10.1038/s41598-020-75209-z (PMC7589531; doi:10.1038/s41598-020-75209-z)
Supplement: Supplementary file 1 — Supplementary Information [file 41598_2020_75209_MOESM1_ESM.pdf]

CHIA SEEDS AS A POTENTIAL COGNITIVE BOOSTER IN THE APP23 ALZHEIMER'S DISEASE MODEL.

Stefanie Schreyer [1,*], Charlotte Klein [1], Anna Pfeffer [1], Justyna Rasińska [1], Laura Stahn [1], Karlotta Knuth [1], Basim Abuelnor [1], Alina Elisabeth Catharina Panzel [1], André Rex [2], Stefan Koch [2,3], Shabnam Hemmati-Sadeghi [1, #], Barbara Steiner [1, #]

[1] Department of Neurology, Charité – Universitätsmedizin Berlin, Freie Universität Berlin, Humboldt-Universität zu Berlin, and Berlin Institute of Health, Charitéplatz 1, 10117 Berlin, Germany

[2] Department of Experimental Neurology and Center for Stroke Research Berlin, Charité – Universitätsmedizin Berlin, Freie Universität Berlin, Humboldt-Universität zu Berlin, and Berlin Institute of Health, Charitéplatz 1, 10117 Berlin, Germany

[3] NeuroCure Cluster of Excellence and Charité Core Facility 7T Experimental MRIs, Charité – Universitätsmedizin Berlin, Freie Universität Berlin, Humboldt-Universität zu Berlin, and Berlin Institute of Health, Charitéplatz 1, 10117 Berlin, Germany

* Corresponding author at: Charité – Universitätsmedizin Berlin, Department of Neurology, Charitéplatz 1, 10117 Berlin, Germany; stefanie.schreyer@charite.de

[#] These authors contributed equally to this work.

Supplemental Table S1: Composition of diets provided by Research Diets Inc. (New Brunswick, NJ, USA).

**Diet**	**CD**	**CD+Chia**	**SRD**	**SRD+Chia**
Product No.	D16022602	D16022603	D16022604	D16022605
kcal/g	4.15	4.15	4.28	4.28
**Ingredient (g)**				
Casein	160.5	104.75	160.5	104.75
DL-Methionine	3.0	3.0	3.0	3.0
Corn starch	442.5	332.41	0	0
Maltodextrin 10	125.0	125.0	25.0	0
Sucrose	0	0	542.5	457.41
Cellulose, BW200	50.0	0	50.0	0
Corn oil	98.4	1.15	98.4	1.15
t-Butylhydroquinone	0.02	0.02	0.02	0.02
Mineral mix S10022M	35.0	35.0	35.0	35.0
Vitamin mix V10037	10.0	10.0	10.0	10.0
Choline bitartate	2.5	2.5	2.5	2.5
Chia seed	0	286.0	0	286.0

Supplemental Table S2: Nutritional facts of chia seeds. Values were obtained from the spec sheet of chia seeds provided by Onset Worldwide LC (Frenchtown, NJ, USA).

**Ingredient**	**g per 100 g**
Protein	19.5 ± 3.9
Carbohydrates	38.5 ± 7.7
Dietary fiber	23 ± 4.6
Fat	34 ± 6.8
thereof saturated fatty acids	3.8 ± 0.7
thereof monounsaturated fatty acids	2.6 ± 0.5
thereof polyunsaturated fatty acids	27.8 ± 5.5
thereof omega-3 fatty acids	21.6 ± 4.2
thereof omega-6 fatty acids	6.2 ± 1.24

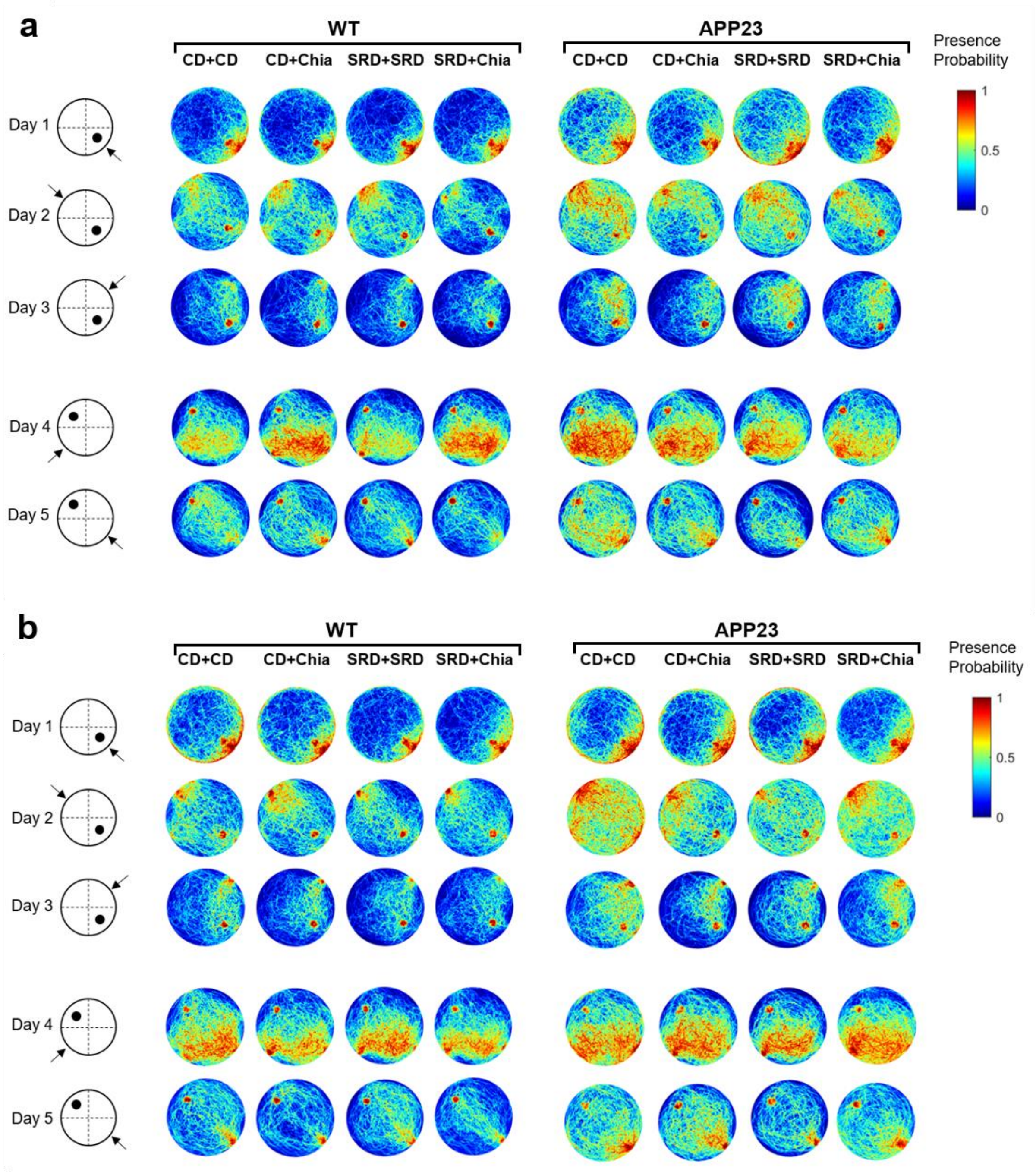

Supplemental Figure S3: Pseudocolor coded heatmaps showing presence probabilities of PRE (a) and ADV mice (b) in the Morris Water Maze. Reddish tones denote high presence probabilities and bluish tones denote low presence probabilities. The left panel indicates the day, the platform position (black dot) and the starting position of mice (arrow). On day 3, APP23 mice on CD+Chia show a more precise search pattern, especially with age. On day 5, APP23 on SRD+SRD display a more distinct search pattern. Abbreviations: WT = wild type control, APP23 = transgenic mouse model, PRE = pre-plaque stage, ADV = advanced-plaque stage, CD = control, SRD = sucrose-rich, Chia = chia seed supplementation.

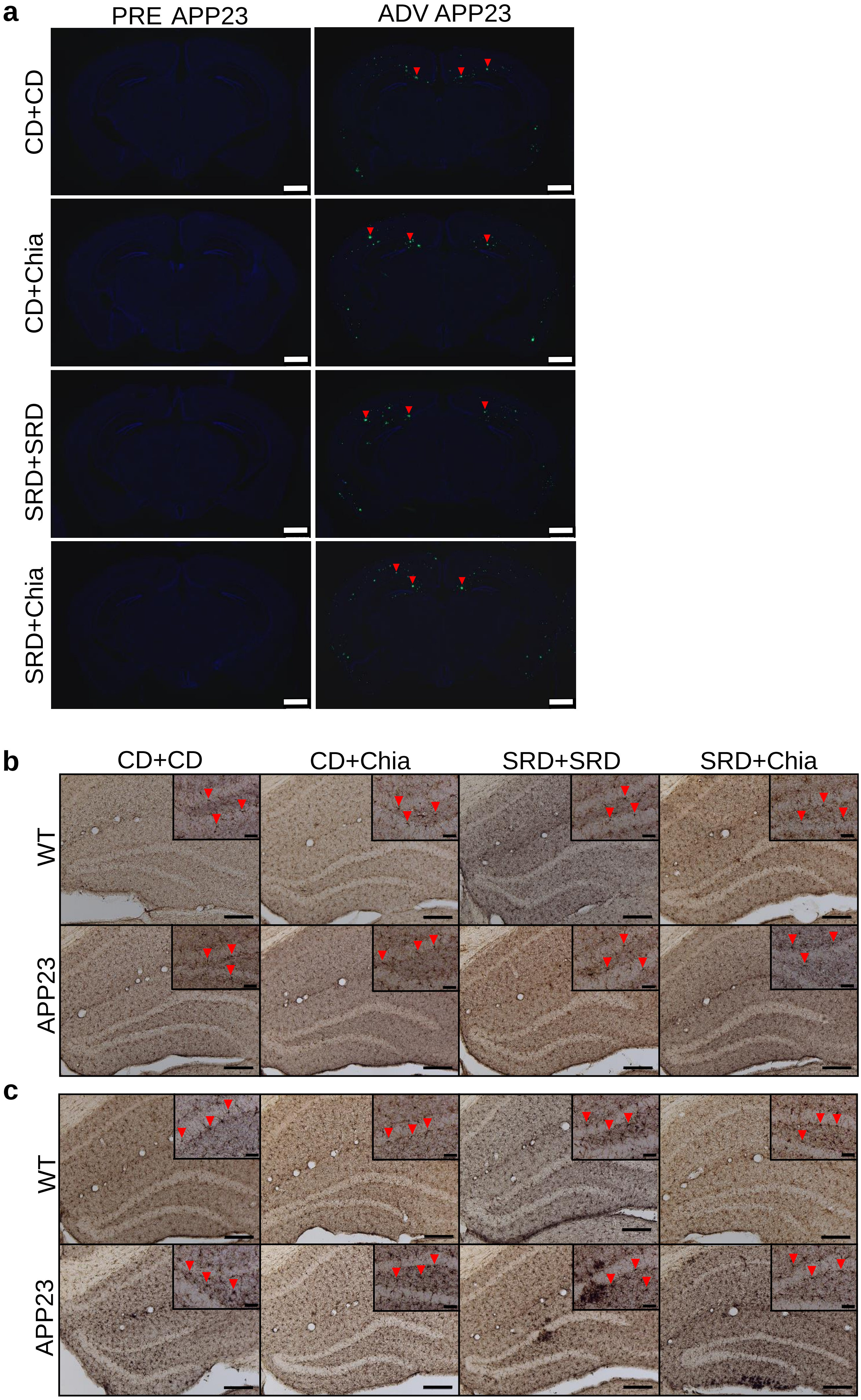
a
PRE APP23
ADV APP23
CD+CD
CD+Chia
SRD+SRD
SRD+Chia
b
CD+CD
CD+Chia
SRD+SRD
SRD+Chia
WT
APP23
c
WT
APP23

Supplemental Figure S4: Representative microscope images of the histological stainings. a: Images of pFTAA staining to visualize Aβ plaques of PRE and ADV mice. Merged pictures of DAPI (blue) and pFTAA (green) fluorescence. Red arrowheads exemplarily mark Aβ plaques. Scale bar = 1 mm. b: Images of Iba1 staining to visualize microglia and macrophages in the hippocampus of PRE (b) and ADV mice (c). Red arrowheads exemplarily mark Aβ plaques. Scale bar (large pictures) = 200 µm, scale bar (close ups in the upper right corner) = 50 µm. Abbreviations: WT = wild type control, APP23 = transgenic mouse model, PRE = pre-plaque stage, ADV = advanced-plaque stage, CD = control, SRD = sucrose-rich, Chia = chia seed supplementation, pFTAA = fluorescent pentameric oligothiophene, DAPI = diamidino-2-phenylindole, Iba1 = allograft inflammatory factor.

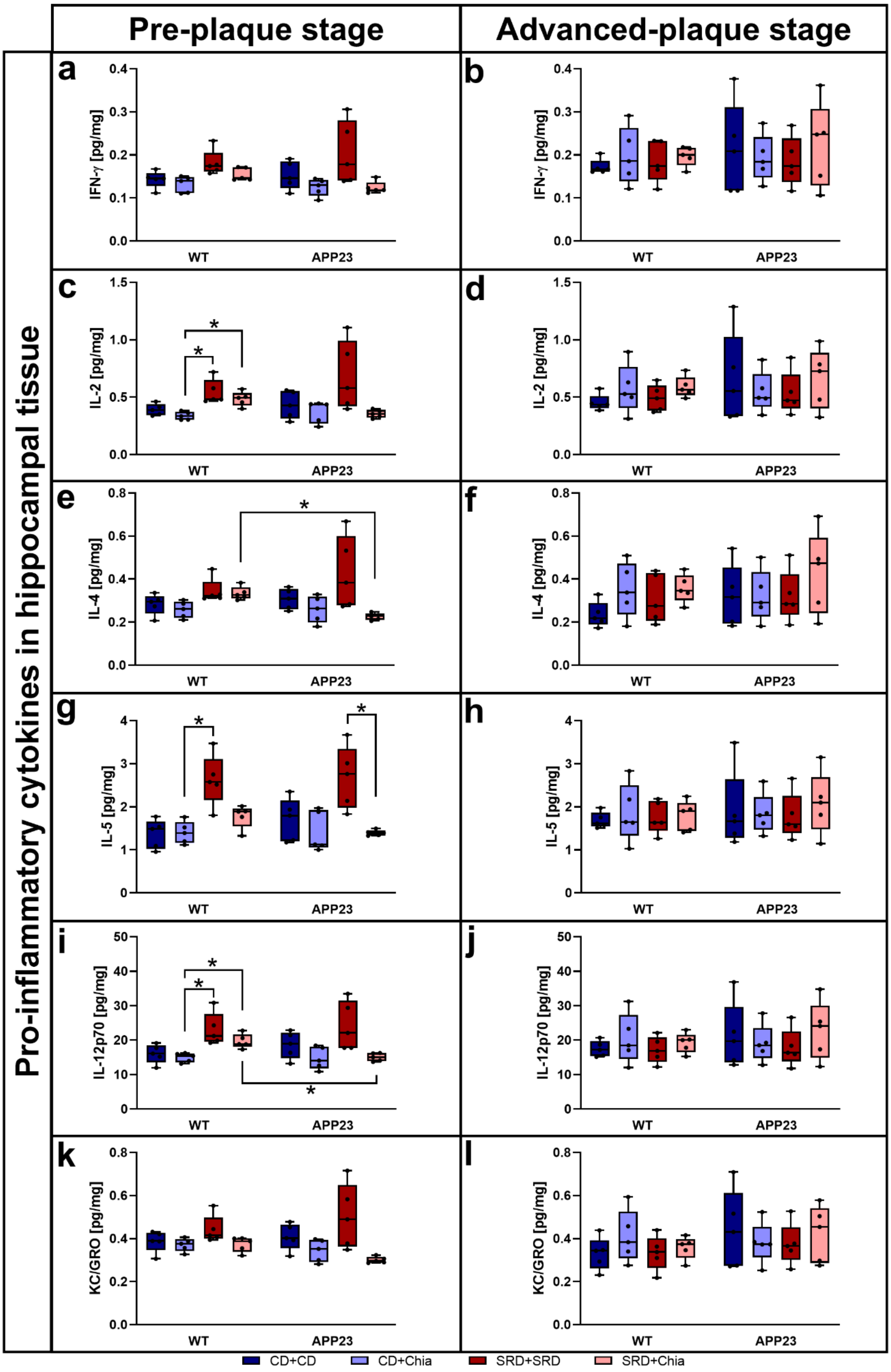

Supplemental Figure S5: Hippocampal levels of pro-inflammatory cytokines. a-b: IFN-γ levels [pg/mg] in the hippocampus of PRE (a) and ADV (b) mice. c-d: IL-2 levels [pg/mg] in the hippocampus of PRE (c) and ADV (d) mice. e-f: IL-4 levels [pg/mg] in the hippocampus of PRE (e) and ADV (f) mice. g-h: IL-5 levels [pg/mg] in the hippocampus of PRE (g) and ADV (h) mice. i-j: IL-12p70 levels [pg/mg] in the hippocampus of PRE (i) and ADV (j) mice. k-l: KC/GRO levels [pg/mg] in the hippocampus of PRE (k) and ADV (l) mice.

Each box represents the 25th to 75th percentile, the line represents the median, whiskers reach from minimum to maximum. An asterisk indicates significant differences between groups regardless of significance level ($p<0.05$), according to nonparametric multiple contrast Tukey-type test. Abbreviations: WT = wild type control, APP23 = transgenic mouse model, PRE = pre-plaque stage, ADV = advanced-plaque stage, CD = control, SRD = sucrose-rich, Chia = chia seed supplementation, IFN-γ = interferon-γ, IL = interleukin, KC/GRO = keratinocyte chemoattractant/human growth-regulated oncogene.

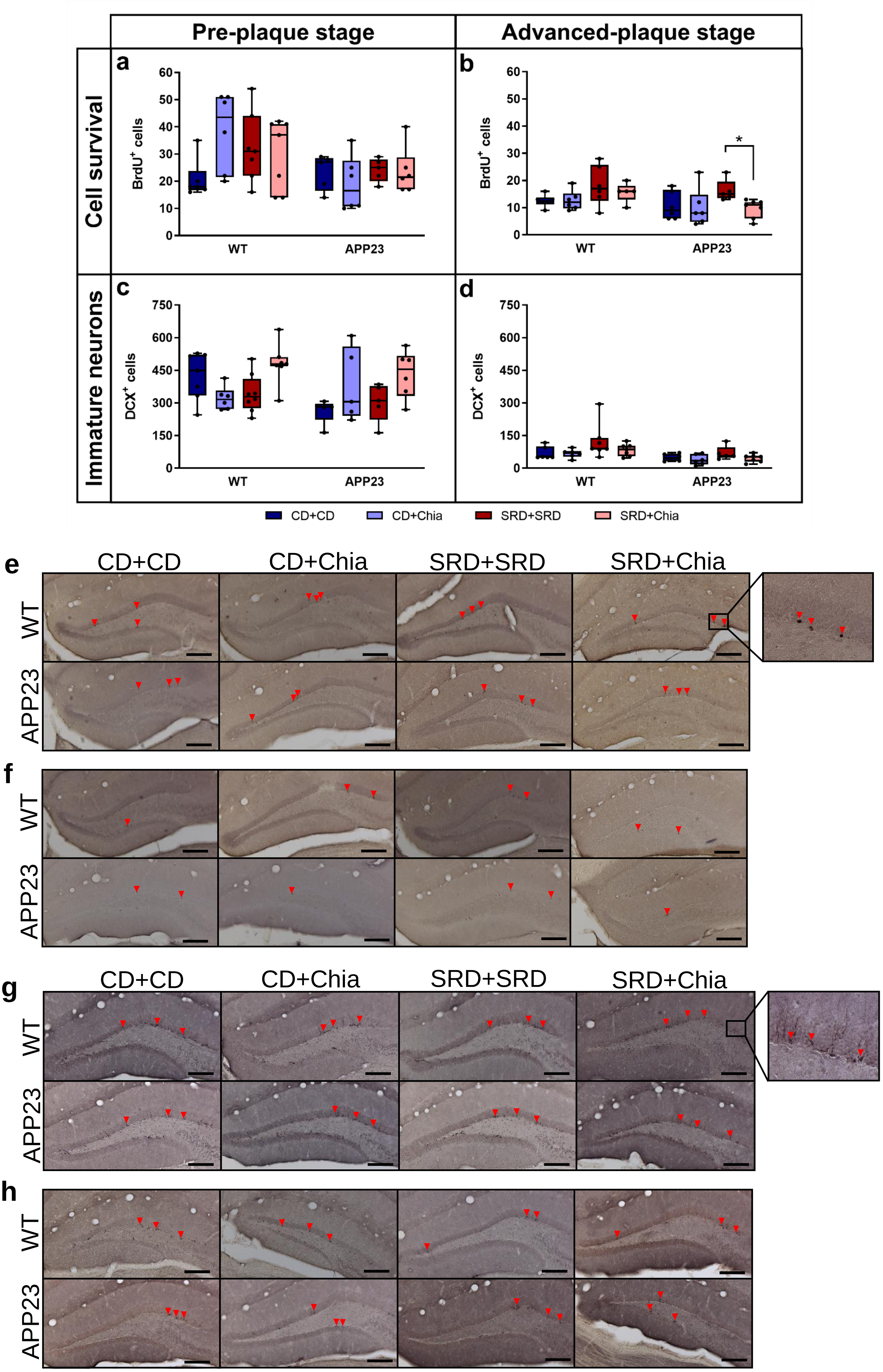
Pre-plaque stage
Advanced-plaque stage
Cell survival
Immature neurons
a
b
c
d
BrdU+ cells
DCX+ cells
WT
APP23
*
CD+CD
CD+Chia
SRD+SRD
SRD+Chia
e
f
g
h

Supplemental Figure S6: Adult hippocampal neurogenesis assessed by survival of BrdU$^+$ cells in PRE (a) and ADV mice (b) and by the number of immature neurons expressing DCX in PRE (c) and ADV mice (d) in the dentate gyrus of the hippocampus. Each box represents the 25th to 75th percentile, the line represents the median, whiskers reach from minimum to maximum. An asterisk indicates significant differences between groups regardless of significance level ($p<0.05$), according to nonparametric multiple contrast Tukey-type test. Representative microscope images of BrdU$^+$ cells of PRE (e) and ADV (f) mice and representative microscope images of DCX$^+$ cells of PRE (g) and ADV (h) mice. Red arrowheads exemplarily mark stained cells. Scale bar = 200 µm. Abbreviations: WT = wild type control, APP23 = transgenic mouse model, PRE = pre-plaque stage, ADV = advanced-plaque stage, CD = control, SRD = sucrose-rich, Chia = chia seed supplementation, BrdU = bromodeoxyuridine, DCX = doublecortin.

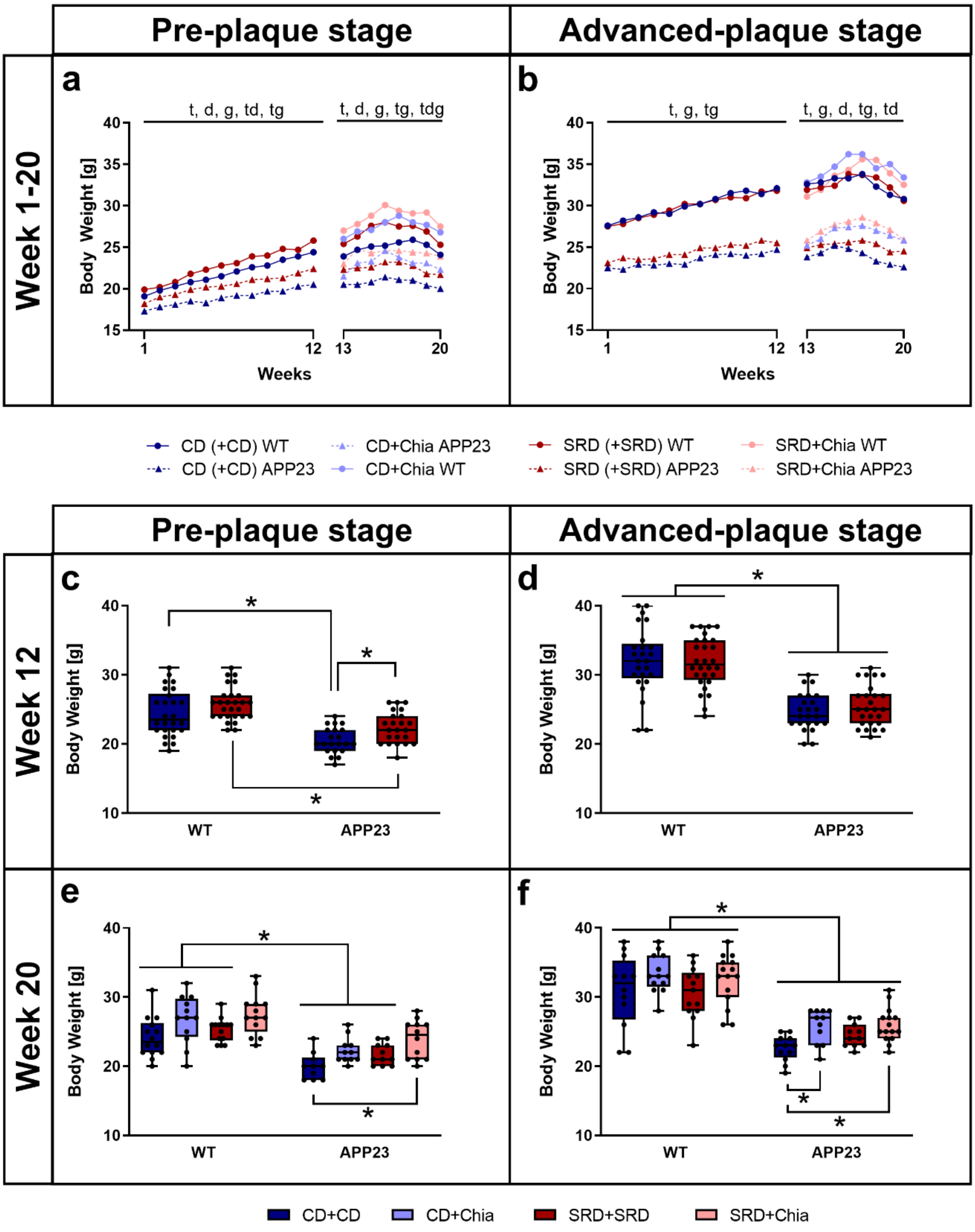

Supplemental Figure S7: Mean body weight [g] over the course of 20 weeks of experiment (a-b), at the end of pretreatment in week 12 (c-d) and at the end of therapy in week 20 (e-f). Letters indicate significant factors (t = time, d = diet, g= genotype, combination of letters = interaction of two or three factors), regardless of significance level ($p<0.05$). Each box represents the 25th to 75th percentile, the line represents the median, whiskers reach from minimum to maximum. An asterisks indicates significant differences between groups, regardless of significance level ($p<0.05$) according to nonparametric multiple contrast Tukey-type test. Abbreviations: WT = wild type control, APP23 = transgenic mouse model, PRE = pre-plaque stage, ADV = advanced-plaque stage, CD = control, SRD = sucrose-rich, Chia = chia seed supplementation.

a: PRE mice significantly gained weight during pretreatment ($F(5.685,82.352)=346.769$, $p<0.001$). Genotype significantly affected body weight of PRE mice ($F(1,82.352)=70.164$, $p<0.001$), so that APP23 mice on average weighed 2.6 g less than WT mice. Diet also significantly influenced body weight in the PRE group ($F(1,82.352)=17.205$, $p<0.001$), such as mice receiving SRD on average weighed 1.3 g more than mice receiving CD. During therapy, time again significantly influenced body weight of PRE mice ($F(5.394,71.940)=38.669$, $p<0.001$). Genotype also significantly affected the body weight in PRE mice during therapy ($F(1,71.940)=71.498$, $p<0.001$), so as to APP23 mice on average weighed 4.3 g less than WT mice. Again, diet significantly altered the body weight PRE mice ($F(2.823,71.940)=8.763$, $p<0.001$).

b: ADV mice also significantly gained weight during pretreatment ($F(7.291,88.686)=93.295$, $p<0.001$). Genotype significantly influenced body weight ($F(1,88.686)=113.178$, $p<0.001$), such as APP23 mice on average weighed 6.0 g less than WT mice. In contrast to the PRE group, diet did not affect body weight in ADV mice. During therapy, time also significantly influenced body weight of ADV mice ($F(5.684,64.711)=52.330$, $p<0.001$). Additionally, genotype significantly affected the body weight in ADV mice ($F(1,64.711)=136.102$, $p<0.001$), such as APP23 mice on average weighed 7.6 g less than WT mice. Diet also significantly influenced the body weight in ADV mice ($F(2.873,64.711)=3.692$, $p=0.012$).

c: In week 12, PRE APP23 mice receiving CD weighed on average 3.9 g less than age-matched WT mice on CD ($p<0.001$). Similarly, body weight of PRE APP23 mice on SRD was on average reduced by 3.3 g compared to age-matched WT mice on SRD ($p<0.001$). However, PRE APP23 mice on SRD showed on average by 1.9 g increased body weight compared to PRE APP23 mice on CD ($p=0.022$), whereas WT showed no change in body weight due to SRD.

d: In week 12, ADV APP23 mice on CD and SRD weighed on average 7.4 g and 6.3 g less than age-matched WT mice on the respective diet (both $p<0.001$). In contrast to PRE mice, SRD induced no weight change in either genotype in ADV mice.

e: In week 20, PRE APP23 mice on CD+CD weighed on average 5.8 g less than age-matched WT mice on either diet ($0.012>p<0.001$). PRE APP23 mice receiving SRD+Chia showed on average by 2.3 g increased body weight compared to PRE APP23 mice receiving CD+CD ($p=0.034$).

f: In week 20, ADV APP23 mice on CD+CD weighed on average 9.2 g less than age-matched WT mice on either diet ($0.009>p<0.001$). CD+Chia and SRD+Chia increased body weight in ADV APP23 mice by 3.2 g and 3.3 g compared to CD+CD ($p=0.046$ and $p=0.008$).

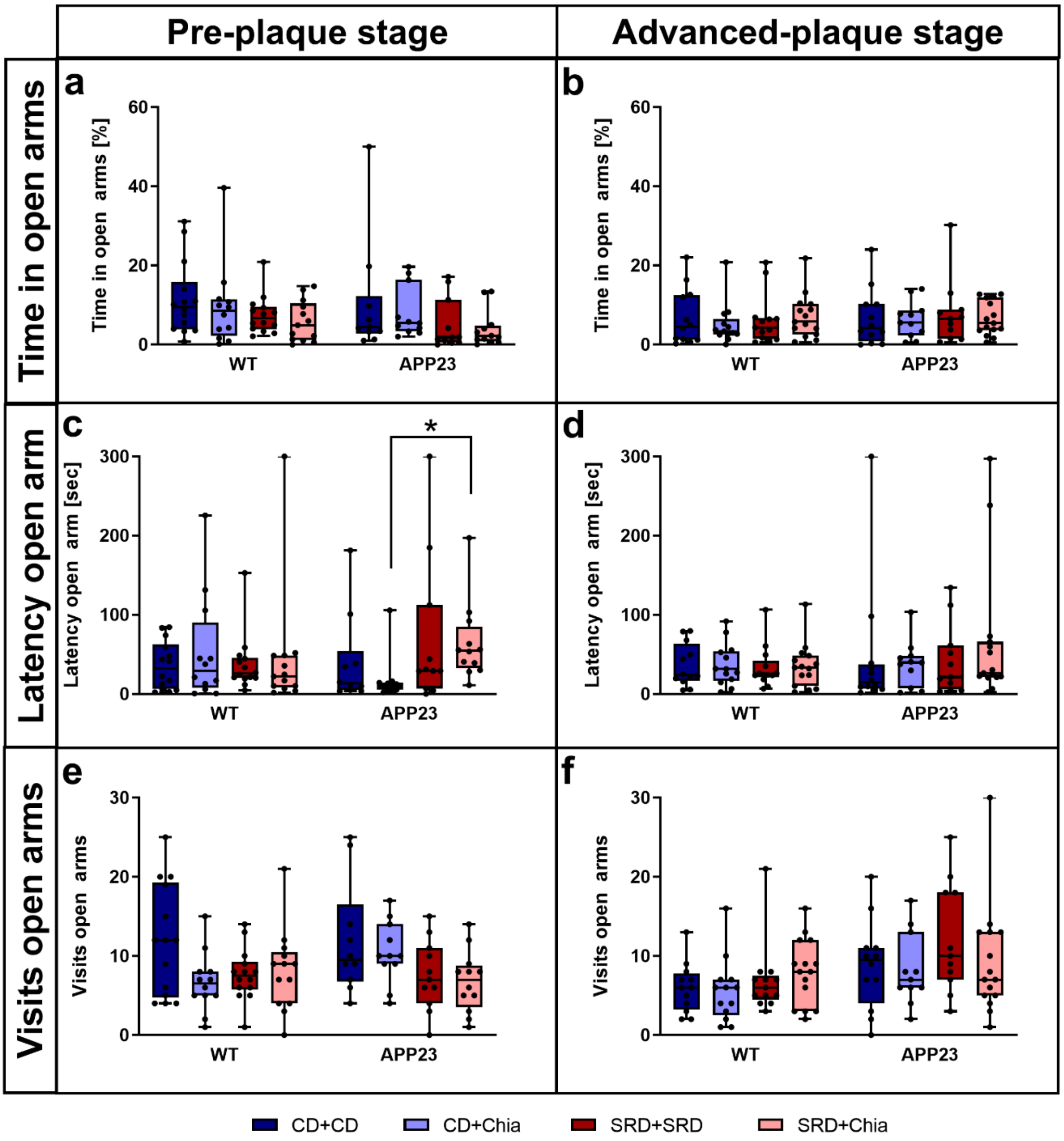

Supplemental Figure S8: Parameters of anxiety examined in the Elevated Plus Maze (EPM) in PRE and ADV mice. Each box represents the 25th to 75th percentile, the line represents the median, whiskers reach from minimum to maximum. An asterisks indicates significant differences between groups, regardless of significance level ($p<0.05$). Abbreviations: WT = wild type control, APP23 = transgenic mouse model, PRE = pre-plaque stage, ADV = advanced-plaque stage, CD = control, SRD = sucrose-rich, Chia = chia seed supplementation.

a-b: Percentage of time PRE (a) and ADV mice (b) spent in the open arms of the EPM. There were no differences between genotypes or dietary groups detectable. c-d: Latency [sec] of PRE (c) and PRE mice (d) of first entry into one of the open arms of the EPM. PRE APP23 mice receiving SRD+Chia showed with 46.2 sec difference a significantly higher latency of the first entry into the open arms than PRE APP23 mice on CD+Chia ($p=0.016$). e-f: Number of visits of PRE (e) and ADV mice (f) of the open arms of the EPM. There were no differences between genotypes or dietary groups detectable.

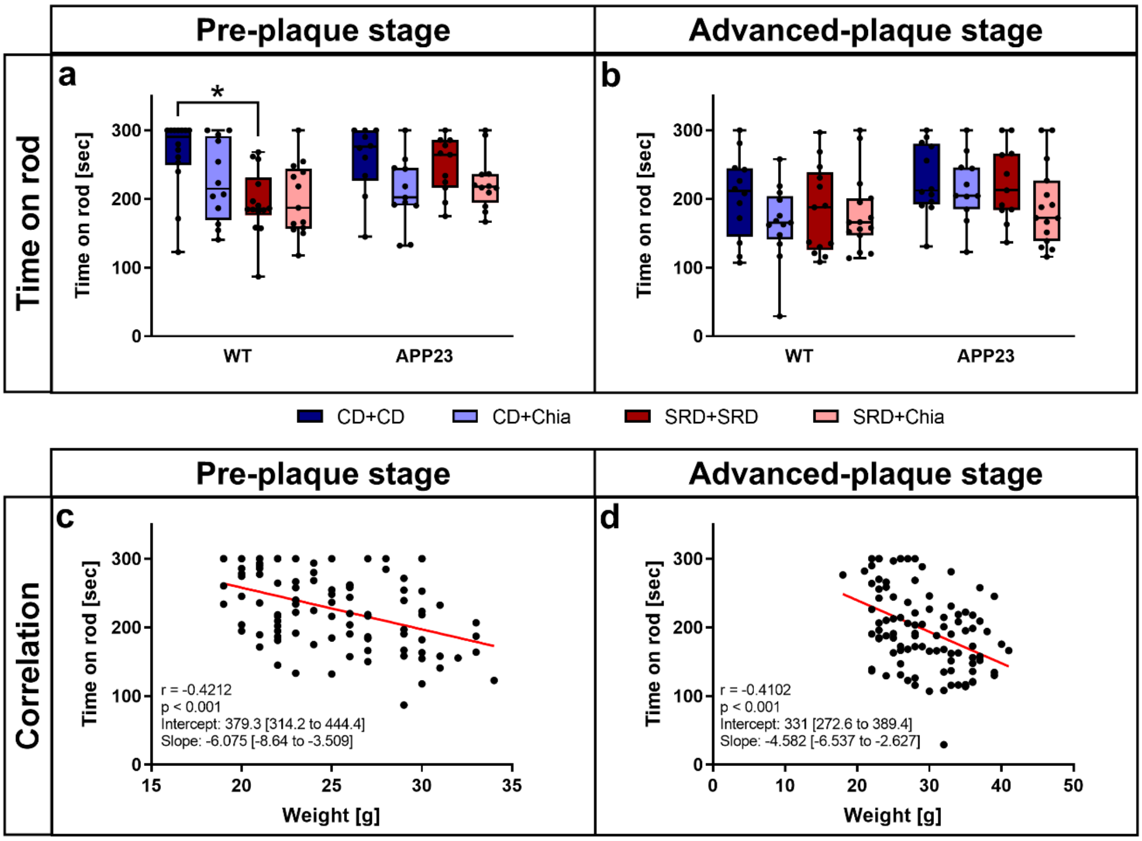

Supplemental Figure S9: Motor coordination and fatigue resistance represented as time PRE (a) and ADV mice (b) spent on the rotating rod and correlation with body weight (c and d). Each box represents the 25th to 75th percentile, the line represents the median, whiskers reach from minimum to maximum. An asterisks indicates significant differences between groups, regardless of significance level ($p<0.05$). Abbreviations: WT = wild type control, APP23 = transgenic mouse model, PRE = pre-plaque stage, ADV = advanced-plaque stage, CD = control, SRD = sucrose-rich, Chia = chia seed supplementation.

a-b: WT mice of the PRE group receiving SRD+SRD stayed with 69.9 sec difference significantly shorter on the rod than WT mice on CD+CD ($p=0.036$). No differences in time on the rod were detectable in ADV mice. C-D: Time on the rod negatively correlated with bodyweight of mice in both age groups, i.e. mice weighing more spent less time on the rod.
